# Immunotherapeutic effects of intratumoral nanoplexed poly I:C

**DOI:** 10.1186/s40425-019-0568-2

**Published:** 2019-05-02

**Authors:** M. Angela Aznar, Lourdes Planelles, Mercedes Perez-Olivares, Carmen Molina, Saray Garasa, Iñaki Etxeberría, Guiomar Perez, Inmaculada Rodriguez, Elixabet Bolaños, Pedro Lopez-Casas, Maria E. Rodriguez-Ruiz, Jose L. Perez-Gracia, Ivan Marquez-Rodas, Alvaro Teijeira, Marisol Quintero, Ignacio Melero

**Affiliations:** 1 Center for Applied Medical Research (CIMA), University of Navarra, Avenida Pio XII, 55, 31008 Pamplona, Spain; 2 Bioncotech Therapeutics S.L, Valencia, Spain; 3 Medical Oncology Department, Hospital General Universitario Gregorio Marañón, Madrid, Spain; 4 Clínica Universidad de Navarra, Pamplona, Spain; 5 CIBERONC, Madrid, Spain; 6 IDISNA, Instituto de investigación de Navarra, Pamplona, Spain

**Keywords:** BO-112, Intratumoral immunotherapy, Nanoplexed poly I:C

## Abstract

Poly I:C is a powerful immune adjuvant as a result of its agonist activities on TLR-3, MDA5 and RIG-I. BO-112 is a nanoplexed formulation of Poly I:C complexed with polyethylenimine that causes tumor cell apoptosis showing immunogenic cell death features and which upon intratumoral release results in more prominent tumor infiltration by T lymphocytes. Intratumoral treatment with BO-112 of subcutaneous tumors derived from MC38, 4 T1 and B16-F10 leads to remarkable local disease control dependent on type-1 interferon and gamma-interferon. Some degree of control of non-injected tumor lesions following BO-112 intratumoral treatment was found in mice bearing bilateral B16-OVA melanomas, an activity which was enhanced with co-treatment with systemic anti-CD137 and anti-PD-L1 mAbs. More abundant CD8^+^ T lymphocytes were found in B16-OVA tumor-draining lymph nodes and in the tumor microenvironment following intratumoral BO-112 treatment, with enhanced numbers of tumor antigen-specific cytotoxic T lymphocytes. Genome-wide transcriptome analyses of injected tumor lesions were consistent with a marked upregulation of the type-I interferon pathway. Inspired by these data, intratumorally delivered BO-112 is being tested in cancer patients (NCT02828098).

**Electronic supplementary material:**

The online version of this article (10.1186/s40425-019-0568-2) contains supplementary material, which is available to authorized users.

## Background

Intratumoral local immunotherapy is gaining interest as a way to broaden the therapeutic window of immunotherapy agents and confine their effects to the tumor microenvironment and tumor-draining lymph nodes (TDLN) [1]. Moreover, a number of examples indicate that following intratumoral release, therapeutic effects against distant disease are observed beyond the injected tumor [1–3]. Immunotherapy agents in the form of cytokines [4, 5], recombinant viruses [6, 7], monoclonal antibodies (mAbs) [8], and pathogen-associated molecular patterns [9–11] can be delivered by intratumoral approaches.

Poly I:C is an analogue of double-stranded viral RNA that acts as an agonist of innate immune receptors deployed to detect infection by such microorganisms. Endosomal TLR3 and intracellular MDA5 and RIG-I may detect the compound leading to upregulation of type-I interferon (IFN-I) and other proinflammatory pathways [12, 13]. Indeed, Poly I:C was originally described for its effects as an exogenous IFNα/β inducer [14] with well documented antiviral and antitumor effects in mice [15, 16]. These include immunotherapeutic efficacy observed following intratumoral injections [16–18] and it has been extensively used as a vaccine adjuvant including cancer vaccines [19–22].

A number of compounds have been produced to exploit the proimmune effects of Poly I:C in the clinic. Among these are Ampligene [23], Hiltonol [24–26] and BO-112 [27]. Hiltonol, a Poly I:C formulation stabilized by poly-L-lysine, is the most advanced of such compounds in the clinic, as it has been tested subcutaneously in healthy volunteers [28] and in cancer patients when given intratumorally [10, 29] and intramuscularly [30]. In healthy volunteers, subcutaneous injection induces prominent transient inflammation and a marked type-I interferon transcriptional signature among circulating PBMC [28]. BO-112 is a nanoplexed form of Poly I:C coupled to polyethylenimine (PEI) reminiscent of BO-110, a previous format of the compound that was found to in-vivo induce apoptosis in melanoma cells as a result of intense autophagy [31, 32].

In this article, we studied the immunotherapeutic profile of BO-112 following intratumoral delivery in experimental models. Our observations include induction of immunogenic cell death in a small fraction of tumor cells and objective immunotherapeutic activity dependent on Interferon-γ (IFNγ) and type-I interferon. Finally, we show the involvement of BATF-3-dependent conventional type-I dendritic cells (cDC1) [33].

## Materials and methods

### Animals and cell lines

Animal studies were approved by the Ethics Committee of Animal Experimentation (CEEA) of the CNB and of the CIMA with compliance with national, institutional and EU guidelines. Six- to eight-week-old female BALB/c and C57Bl/6 were purchased from Envigo (www.envigo.com). C57Bl/6 *Batf3*^*tm1Kmm/J*^ (Batf3^−/−^) [33] and *IFN-a/bR*^*o/o*^ (IFNARKO) [34] were kindly provided, by Dr. Kenneth M. Murphy, Washington University, St. Louis, MO and by Matthew Albert (Institut Pasteur, Paris) respectively, and were bred at CIMA in specific pathogen-free conditions. Mice were housed in the Animal Facility of Centro Nacional de Biotecnologia (CNB-CSIC, Madrid, Spain) and Centro de Investigacion Medica Aplicada (CIMA, Pamplona, Spain).

B16-F10 mouse melanoma cells and 4 T1 mouse breast carcinoma were purchased from the ATCC, and B16-OVA melanoma cells and MC38 colon carcinoma cells were a kind gift from Dr. Lieping Chen (Yale University, New Heaven, CT) and Dr. Karl E. Hellström (University of Washington, Seattle, WA) respectively. Tumor cells were cultured in RPMI 1640 (Gibco) containing 10% fetal bovine serum (FBS, Sigma-Aldrich), 2 mM glutamine (Gln, Gibco), 100 U/ml penicillin and 100 μg/ml streptomycin (100 U/ml), and 50 μM 2-mercaptoethanol (Gibco). The B16-OVA cell line was supplemented with 400 μg/mL Geneticin (Gibco). Cell lines were routinely tested for mycoplasma contamination (MycoAlert Mycoplasma Detection Kit, Lonza).

UMBY and ICNI human melanoma were derived from primary surgical samples of metastatic lesions of patients at the Department of Dermatology, University Hospital Erlangen and grown in DMEM (Gibco) containing 10% FBS, 4 mM Gln and 1% P/S. HT-29 and HCT 116 colon cancer from the ATCC were cultured in RPMI, 2 mM Gln, 10% FBS and 1% P/S. SK-BR-3 and BT-474 breast cancer cell lines were a kind gift from Dr. López-Botet, IMIM, Barcelona and were grown in DMEM/F12 (1:1) (Invitrogen), containing 2.5 mM Gln, 10% STF and 1% P/S.

### BO-112

BO-112 was developed and provided by Bioncotech Therapeutics (Madrid, Spain). All experiments were performed with the same batch.

### In vitro experiments

The in vitro cytotoxicity of BO-112 in mouse and human cell lines was continuously assessed by measuring electric impedance in an xCELLigence machine (ACEA). Tumor cells (1.5-2 × 10^5^) were seeded on specific 8-well plates to measure electric impedance. After 4-5 h, BO-112 or Poly I:C (Sigma) was added to culture media at identical concentrations in a final volume of 200 μL per well. PEI (Polyplus-transfection®) was added to culture media at the same concentrations as it is present in BO-112 formulation. Electric impedance was measured every five minutes for 48 h. In vitro BO-112 cytotoxicity was also assessed by the CellTiter AQueous One Solution Cell Proliferation Assay (MTS, Promega). Briefly, tumor cells (5 × 10^3^ cells/well; 96 flat-well plates, 8 replicates per condition) were cultured for 48 h, alone or with BO-112 (0.25, 0.5 and 1 μg/mL) and absorbance (OD 492 nm) measured in an ELISA plate reader. Three independent MTS assays were performed. Cell viability is referred to untreated cells (100%).

For RNA expression analyses, B16-OVA cell lines were cultured 24 h with BO-112 at 0.5 μg/mL or in the presence of equivalent volumes of BO-112 vehicle.

HMGB1 detection in culture supernatants was performed with HMGB1 ELISA detection kit following the manufacturer’s instructions (IBL International ST51011).

### In vivo experiments

B16-F10 and B16-OVA melanoma, MC38 colon carcinoma or 4 T1 breast carcinoma cells were injected subcutaneously (5 × 10^5^–10^6^) into the right flank of 8- to 10-week-old female C57BL/6 or BALB/c (6–11 mice/group) on day 0. Tumors were measured twice per week with calipers and the volume calculated (length x width^2^/2). When tumors reached a volume of 80–100 mm^3^ (day 0) mice were randomized into different groups of treatment according to the experiment. Poly I:C or BO-112 formulation (2.5 mg/Kg, 100 μl), was administered by intratumoral injection twice per week for three weeks (six doses in total). The control group received intratumoral injections of 5% glucose (BO-112 vehicle, identical volume) or PEI (identical amount per dose as present in each dose of BO-112). Survival was monitored daily, and tumors were measured twice per week until the animals died or the tumor volume reached the maximum allowed size.

To evaluate the systemic antitumor effects, 5 × 10^5^ (injected/treated tumor) and 1.5 × 10^5^ (contralateral tumor) B16-OVA cells were injected into each flank respectively. For evaluation of cDC1 in BO-112-meditated antitumor response, identical experiments were performed in Batf3^−/−^ mice (in parallel with WT mouse groups). For evaluation of intratumoral BO-112 in combination with systemic immunostimulatory monoclonal antibodies, mice were intratumorally injected with BO-112 or vehicle. The intratumoral treatment schedule was the same as that described for single tumor models. Starting at the second BO-112 administration, mice received concomitant intraperitoneal administration (150 μg/dose) of either InVivoPlus anti-PD-L1 (10F.9G2), InVivo anti-CD137 (3H3) or InVivoMAb rat IgG from BioXCell.

For flow cytometry, IHC and RNA extraction experiments, mice received two intratumoral administrations and were euthanized 48 h post-second dose.

### Flow cytometry

Fresh tumors were excised from mice, weighed and mechanically dissected and enzymatically digested for 15–30 min at 37 °C with appropriate medium: DMEM F-12 (Gibco), 1 mg/mL Collagenase 1A (Roche), 2.5 U/mL Dispase (Roche), 20 U/mL DNAse-I (Roche), 20 mM HEPES (Lonza) and antibiotics. The enzymatic reaction was stopped with 5% FBS in phosphate-buffered saline (PBS). After hypotonic lysis, cell aggregates were removed by filtering the cell suspension with a 70-μm cell strainer (BD Falcon, BD Bioscience) and counted. Lymph nodes were excised from mice and mechanically disrupted and passed through a 70-μm cell strainer. Perfect count microspheres (Cytognos) were used as an internal standard according to the manufacturer’s instructions to calculate absolute cell counts in cell suspensions.

Single cell suspensions from tumors and lymph nodes were previously treated with FcR-Block (anti-CD16/32 clone 2.4G2; BD Biosciences Pharmingen) and were then surface stained at 4 °C with fluorochrome-labeled antibody cocktails defined for each staining. Tetramer staining was performed according to the manufacturer’s protocol. Flow cytometry antibodies, tetramers, cell death stainings and isotype controls are listed in Additional file 1. Table S1. For intracellular FOXP3 staining, cells were fixed and permeabilized using the True-Nuclear™ Transcription Factor Buffer Set (Biolegend).

Samples were acquired in a Gallios Cytometer (Beckman Coulter), a FACSCanto II (BD Biosciences) and a CytoFLEX Flow cytometer (BD Biosciences). Kaluza Flow Analysis Software (Beckman Coulter) and FlowJo (Treestar) software were used for data analysis.

### Depletion experiments

100–300 μg/dose of anti-CD4 (GK1.5), anti-CD8 (2.43), anti-NK1.1 (PK136), anti-Gr1 (RB6-8C5) mAbs or Rat IgG2b (LTF-2) from BioXCell, were injected one day before therapy, concurrently with the first intratumoral injection and at days 3, 7, and 10 after the beginning of therapy. Cell depletion was validated in blood samples by flow cytometry analysis, showing a specific reduction of more than 95% of each respective cell subset. Gr1 depletion was confirmed in the tumor microenvironment on day 7 (the day of the first BO-112 injection). For IFNγ neutralization, mice were treated with 250 μg of anti-IFNγ (XMG1.2) or rat IgG the day prior to each BO-112 treatment. Then, mice were injected weekly with for depletion maintenance (100 μg/dose).

### Tissue histology and immunostaining

Formaldehyde-fixed and paraffin-embedded tissue sections (3 μm thick) were cut, dewaxed and hydrated. Heat induced antigen retrieval was applied for 30 min at 95 °C in 0,01 M Tris-1 mM EDTA buffer (pH = 9) in a Pascal pressure chamber (S2800, Dako). Sections were incubated overnight at 4 °C with anti-CD4 (1:1000; Abcam, ab183685) or anti-CD8 (1:300; Cell Signaling, 98,941) Visualisation was performed using MACH 2 rabbit AP-polymer (Biocare Medical, RALP525) with StayRed (Abcam, ab103741) as chromogen according to the manufacturer’s instructions.

### RNA extraction

Total RNA was isolated in two steps using TRIzol (Life technologies) and Rneasy Mini-Kit (Quiagen) purification, following the manufacturer’s RNA cleanup protocol. The assessment of RNA integrity was performed with the Agilent 2100 bioanalyzer (Agilent Technologies) and high-quality RNA was hybridized to Affymetrix Clariom S Mouse Affymetrix microarrays following the manufacturer’s protocol.

### Gene expression analysis

The transcriptome experiment with Clariom S Mouse Affymetrix microarrays was normalized using the Robust Multichip Average (RMA) algorithm [35]. After quality assessment, a filtering process was performed to eliminate low expression probe sets. Applying the criterion of an expression value greater than 4 in at least 3 samples of one of the experimental conditions (BO-112 or control samples), 21,731 probe sets were selected for statistical analysis in the in vivo experiment. Regarding the differential expression analysis upon BO-112 incubation of B16-OVA in vitro*,* 18,412 probe sets were selected for statistical analysis after applying the criterion of an expression value greater than 4 in at least 2 samples of one of the experimental conditions (BO-112 or control samples).

Linear Models for Microarray Data (LIMMA) [36] was used to identify the probe sets that showed significantly differential expression between experimental conditions. Genes were selected as significant using a B-statistic cut-off (B > 0). R and Bioconductor were used for preprocessing and statistical analysis [37].

The functional enrichment analysis was performed using Ingenuity Pathway Analysis (Ingenuity Systems, www.ingenuity.com), whose database includes manually curated and fully traceable data derived from literature sources. In addition, enrichment analyses of some gene sets of interest extracted from different publications [38, 39] were performed using the hypergeometric distribution in R [37]. Microarray expression data can be downloaded from Gene Expression Omnibus (GEO) under the Series accession number GSE116078.

### Statistical analysis

Statistical analyses were performed using Prism software (GraphPad Software, Inc.). A two-tailed Student’s t-test or Mann–Whitney tests were used to analyze statistical differences between groups. The Mantel-Cox test was used for survival analysis. For tumor growth data analyses, mean volumes of tumors over time were fitted using the formula y = A x e (t-t0) / (1 + e(t-t0)/B), where t represents time, A the maximum size reached by the tumor and B its growth rate. Treatments were compared using the extra sum-of-squares F test. Values of *p* < 0.05 (*), *p* < 0.01 (**) and *p* < 0.001 (***) were considered significant.

## Results

### Intratumoral BO-112 controls transplanted syngeneic tumors and induces cell death in a fraction of malignant cells

BO-112 is a GMP-grade pharmaceutical composition of nanoplexed Poly I:C (300–5000 mer) coupled to polyethylenimine characterized by their monomodal diameter distribution (at least 90% of particles mono-modal diameter distribution bellow 300 nm) and z-average diameter (less or equal 150 nm) (PCTEP2016078078, Additional file 2: Figure S1). Previous forms of Poly IC-PEI nanocomplexes termed BO-110 have been intravenously delivered in mouse models giving rise to therapeutic effects against subcutaneous transplanted melanomas in a fashion related to its ability to induce tumor cell apoptosis in the context of intense autophagy elicited via MDA5 stimulation in malignant cells [31, 32].

**Fig. 1:**
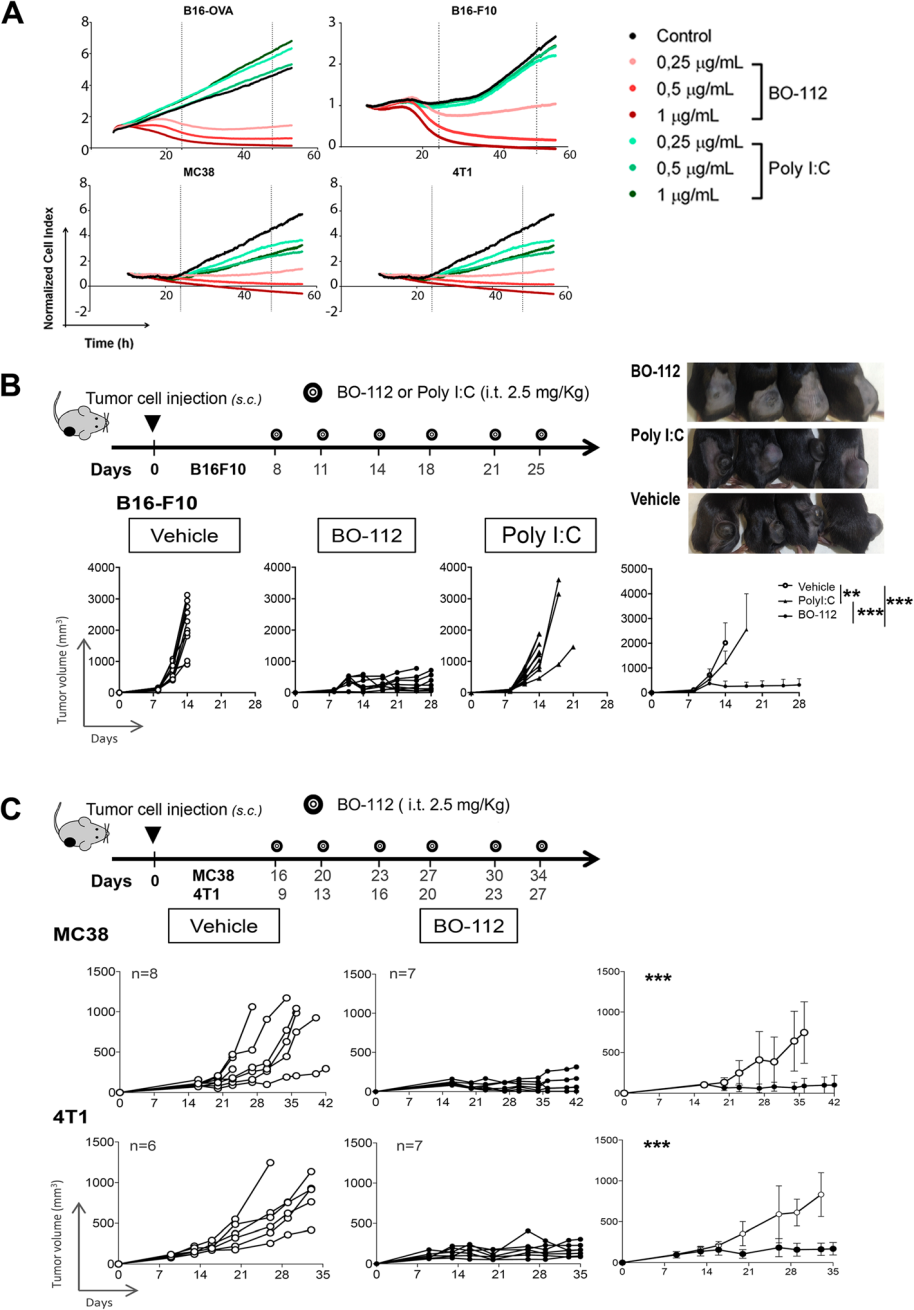
Local injection of BO-112 exerts antitumor effects. **a**. Cell viability (in terms of electric impedance) of cultured tumor cell lines was measured in xCELLigence plates over time in the presence of different concentrations of BO-112 or Poly I:C as indicated, to study effects on cell viability. **b**. Tumor volume follow-up of in vivo engrafted syngeneic B16F10 tumors treated intratumorally with control vehicle, Poly I:C or BO-112 as indicated in the diagram. Representative photographs of mice treated with BO-112, Poly I:C or control vehicle are included as an inset. **c**. Individual follow-up of tumor volume means ± SD (in graphs on the right) of MC38 and 4 T1-bearing mice treated with BO-112 or control vehicle as indicated. Experiments are representative of two similarly performed. ***P < 0.001

In keeping with those findings, Fig. 1a shows that BO-112 induces death in a dose-dependent manner in cultured cell lines able to engraft in syngeneic mice representing melanoma (B16-F10, B16-OVA), colon cancer (MC38) and triple negative breast cancer (4 T1). Tumors derived from such cell lines are described as poorly immunogenic and difficult to treat with immunotherapy [40]. BO-112-induced cytotoxicity was also observed in a panel of human cell lines (Additional file 3: Figure S2A), including melanoma, colon cancer and breast cancer. Of particular importance, identical concentrations of Poly I:C were not cytotoxic in either mouse or human tumor cell lines (Fig. 1a and Additional file 3: Figure S2A). These results were further confirmed by measuring cytotoxicity in MTS assays in mouse and human cell lines (Additional file 3: Figure S2B).

To study the effects of intratumoral injection in subcutaneous malignant nodules derived from these tumor-cell lines upon engraftment in syngeneic mice, BO-112, Poly I:C or vehicle were repeatedly delivered intratumorally when tumors reached a volume of 80–100 mm^3^ (Fig. 1b and c). Figure 1b shows the very clear therapeutic effects of BO-112 at halting and delaying B16-F10 tumor progression, that were not seen in the mice randomized to receive injection of either Poly I:C or vehicle. Representative photographs showing B16-OVA tumors at day 15 are shown in Fig. 1b (inset). BO-112 antitumor therapeutic effects were also observed in MC38- and 4 T1-tumor bearing mice upon a similar repeated intratumoral treatment regimen with BO-112 (Fig. 1c).

**Fig. 2:**
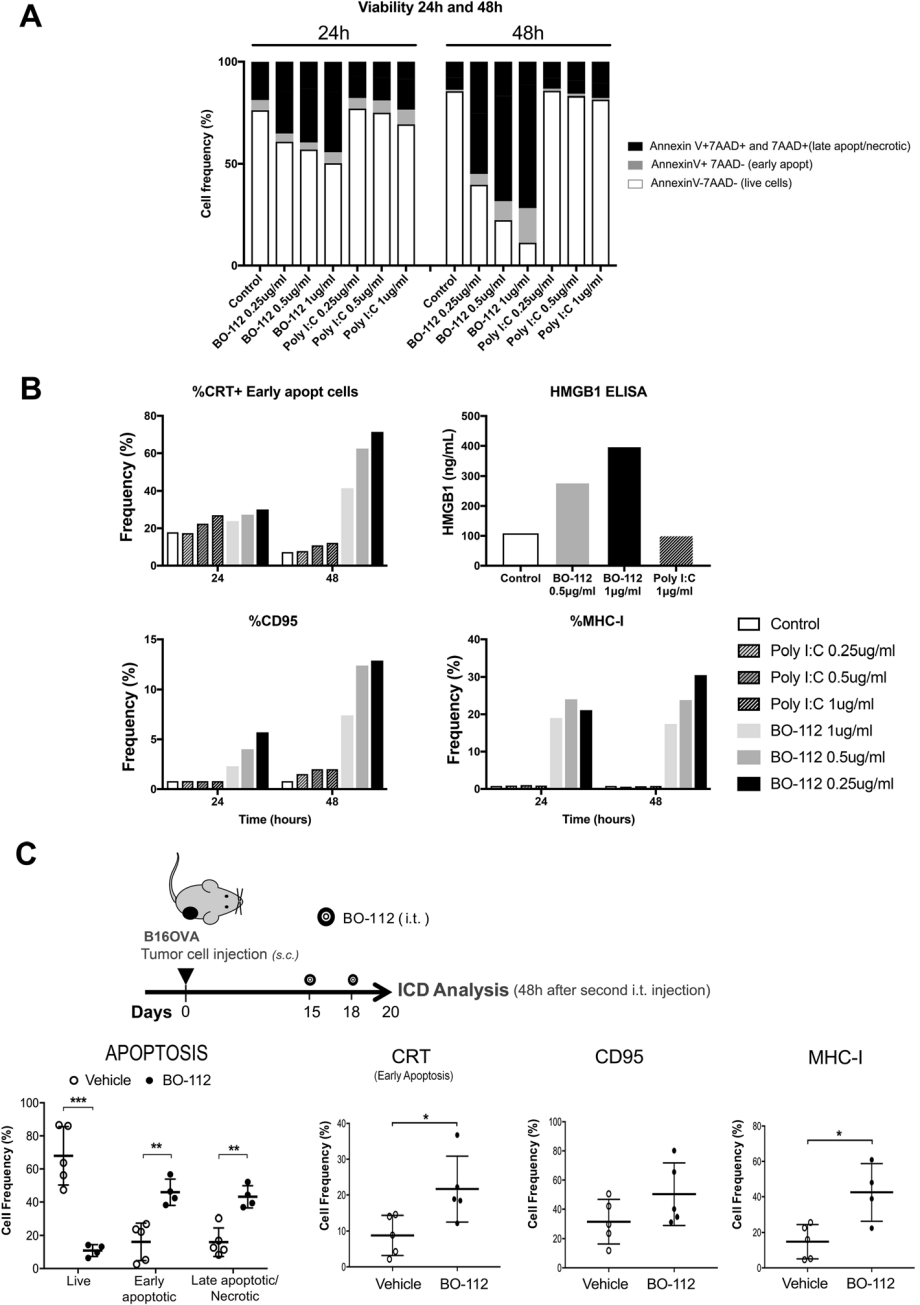
BO-112 induces immunogenic cell death. The characterization of tumor cell death (apoptosis, necrosis, immunogenic cell death) induced by BO-112 was investigated in vitro and in vivo. **a**. and **b**. B16-OVA cells (10^5^ cells/well) were cultured alone or with BO-112 or Poly IC (0.25, 0.5 and 1 μg/ml), for 24 and 48 h. **a**. Apoptosis and necrosis were analyzed by flow cytometry upon staining with Annexin V and 7AAD. **b**. Immunogenic cell death (ICD) hallmarks were analyzed by flow cytometry studying cell surface expression of MHC-I, CD95 and Calreticulin and by measuring HMGB1 release. **c**. B16-OVA tumor bearing mice were intratumorally treated with BO-112 or vehicle (*n* = 5 per group). The diagram shows the schedule of the experiment. Graphs show that intratumoral administration of BO-112 leads to a significant increase in tumor cell apoptosis and necrosis (left) and also promotes the expression of ICD-associated markers on tumor cells. *P < 0.05, **P < 0.01***P < 0.001

Next, we examined whether BO-112 could induce local tumor cell death in an immunogenic fashion [41, 42]. In B16-OVA cultures, tumor cell death was abundant 24 and 48 h after exposure to BO-112 in the form of apoptosis characterized by Annexin V binding and loss of plasma membrane integrity (7AAD staining), as shown in Fig. 2a. This increase in apoptotic cell subsets was also observed in BO-112-treated human cell lines (Additional file 3: Figure S2C). Interestingly, dying or dead cells showed molecular features associated with immunogenic cell death [42] including calreticulin (CRT) exposure on the outer leaf of the plasma membrane, and HMGB1 release to the culture supernatant and CD95 surface expression (Fig. 2b). Such cells also showed enhanced surface expression of MHC class I. Importantly, identical concentrations of Poly I:C added to the culture media failed to induce these hallmarks of immunogenic cell death (Fig. 2b) in tumor cells.

When treating subcutaneous tumors derived from B16-OVA (Fig. 2c), a fraction of apoptotic cells could be observed five days after the onset of intratumoral treatment. Interestingly, in CD45- cells obtained from the tumor nodules there was an increased surface expression of MHC-I, CRT and CD95 (Fig. 2c), suggesting features of immunogenic cell death in vivo. Of note, PEI itself (without Poly I:C) was not cytoxic *in culture* and did not affect progression of B16-OVA melanomas following repeated intratumoral administration in the same quantities as those present in BO112 (Additional file 4: Figure S3 A-C).

### Intratumoral administration is required for antitumor activity as opposed to subcutaneous delivery

Since direct intratumoral injection of BO-112 therapeutically controlled tumor progression, we tested whether similar results could be achieved by subcutaneous delivery elsewhere in the mouse. Such experiments comparing intratumoral versus subcutaneous delivery at the same doses were performed in MC38 and B16-OVA tumor-bearing mice (Additional file 5: Figure S4). As shown in Additional file 5: Figure S4, while intratumoral delivery controlled tumor progression, this was not the case for subcutaneous administrations which failed to control tumor progression. Accordingly, intratumoral release is more efficacious than subcutaneous release under identical experimental conditions.

### Intratumoral BO-112 increases CD8^+^ tumor infiltrating lymphocytes and CD8/Treg ratios

**Fig. 3:**
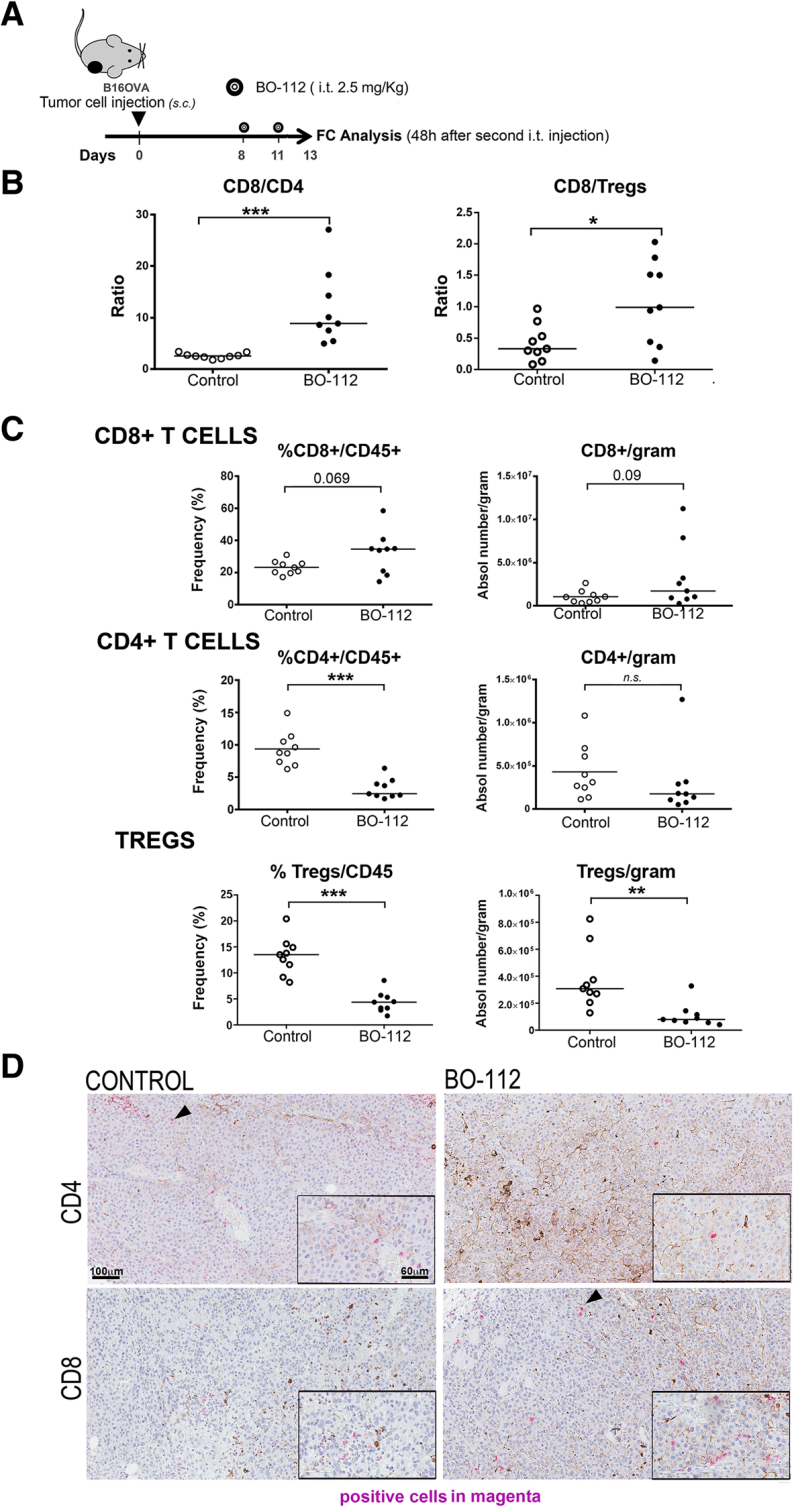
BO-112 intratumoral injection enhances T lymphocyte infiltrates. **a**. Schematic representation of the experiments to surgically harvest tumors following treatment to generate cell suspensions that were analyzed by flow cytometry. **b**. CD8/CD4 and CD8/Treg ratios in cell suspensions. **c**. Percentage of CD8^+^, CD4^+^ and CD25^+^FOXP3^+^ over total intratumoral CD45^+^ leukocytes and absolute numbers per gram of tumor tissue. **d**. Representative microphotographs of CD4 and CD8 immunohistochemistry analyses of sections derived from B16-OVA tumors treated as indicated. Scale bar of the main microphotograph: 100 μm. Scale bar of the inset: 60 μm. Positive cells are stained in magenta. *P < 0.05, **P < 0.01***P < 0.001

Intratumoral BO-112 therapeutic effects could be the result of direct tumor cell death or the result of enhanced antitumor immune responses or a combination of both factors. To start addressing this question, we studied the composition of the lymphoid infiltrates following BO-112 intratumoral delivery in B16-OVA-derived tumors (Fig. 3a and Additional file 6: Figure S5). Interestingly, we observed changes in the leukocyte tumor microenvironment in favor of CD8^+^ cells in proportion to CD4^+^ T cells and regulatory T cells (Tregs) (Fig. 3b). In fact, higher numbers of CD8^+^ cells could be observed per gram of tumor tissue, whereas FOXP3^−^CD4^+^ and FOXP3^+^CD25^+^CD4^+^ Tregs cells decreased (Fig. 3c). These infiltrates were evenly distributed in the tumors as representative microphotographs in Fig. 3d show.

### Efficacy of intratumoral BO-112 given unilaterally to bilaterally tumor-bearing mice in conjunction with systemic anti-CD137 and anti-PD-L1 monoclonal antibodies

**Fig. 4:**
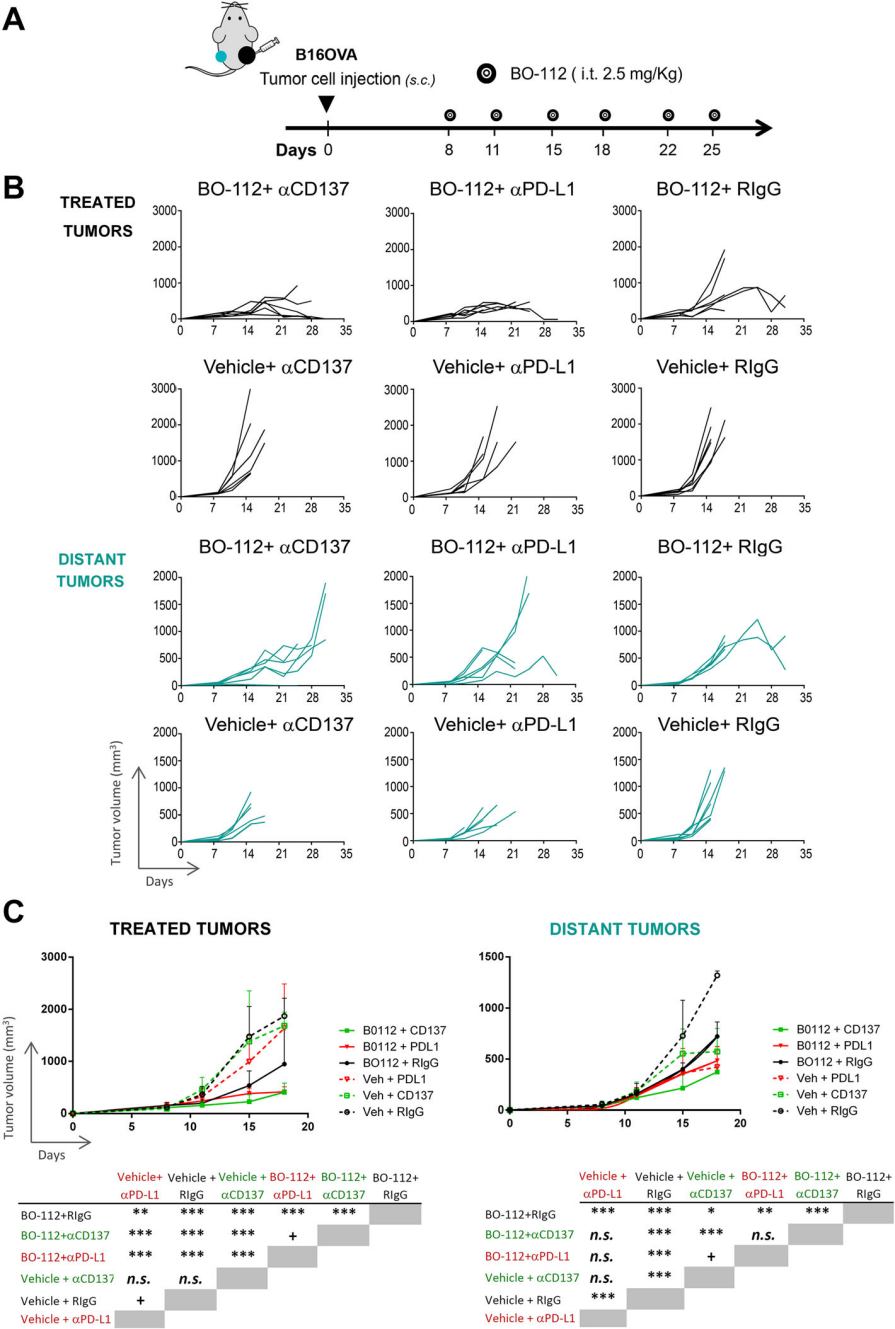
Immunotherapeutic effects of combinations of intratumoral BO-112 with systemic anti-CD137 or anti-PD-L1 monoclonal antibodies. **a**. Schematic representation of experiments in mice bearing two B16-OVA-derived tumors engrafted on opposite flanks and intratumorally treated with BO-112 only in the right lesion and with intraperitoneal administrations of immunomodulatory monoclonal antibodies as indicated. **b**. Tumor volume follow-up of the injected and distant tumors in the different groups of treatment. **c**. Mean ± SD summary indicating statistical significance of the listed comparisons. *P < 0.05, **P < 0.01***P < 0.001

The intratumoral route in all detectable tumors might be possible in oligometastatic patients, but impossible in most advanced cancer patients. Furthermore, microscopic metastatic disease will not be amenable to intratumoral treatment. Therefore, we performed experiments in B16-OVA tumor-bearing mice in which only one of the subcutaneously engrafted tumors was treated (Fig. 4a). These mice were intraperitoneally co-treated with control antibody or immunomodulatory mAbs agonistic for CD137 [43] or antagonistic for PD-L1 [44]. As seen in Fig. 4b and c, BO-112 exerted clear local control of the disease as compared to vehicle. Such local control was enhanced to some extent by both anti-CD137 and anti-PD-L1 mAbs, which did not show any meaningful therapeutic activity by themselves, as shown in their combination with intratumoral control vehicle.

When examining distant tumors (non-injected with BO-112), some degree of tumor-growth control by BO-112 was observed with death of mice being postponed for approximately 1–2 weeks (Fig. 4b and c). Furthermore, when systemic anti-CD137 mAb was used such distant tumor control was further enhanced, although not significantly in the case of anti-PD-L1-treated mice. In this difficult-to-treat melanoma model, our data argue in favor of systemic antitumor activity that might be potentiated by combinations with other immuno-oncology agents.

In B16-OVA tumors intratumorally treated with BO-112, we were able to observe increases in the expression levels of the targets for immunomodulatory mAbs on tumor-infiltrating T cells 48 h following the second BO-112 administration. PD-1 expression markedly rose on CD8^+^ T cells, while CD137 expression was also increased, albeit to a lesser extent (Additional file 7: Figure S6A). Curiously, CD137 was clearly upregulated on NK lymphocytes retrieved from treated tumors. Moreover, PD-L1 levels of surface expression increased on T cells (CD8^+^ and CD4^+^), as well as on NK cells (Additional file 7: Figure S6A). Therefore BO-112 intratumoral treatment at least locally upregulates the targets for the mAbs used in the immunotherapy combinations, including CD8^+^ T cells that co-expressed both PD-1 and CD137 on their surface (Additional file 7: Figure S6B).

In MC38-derived tumors, BO-112 intratumor injections clearly induced an enrichment of PD1^+^, CD137^+^ and double positive CD137^+^PD-1^+^ among CD8+ T cells, although total CD8+ were not augmented (Additional file 8: Figure S7A and B). In line with these findings, in B16-OVA-derived tumors, FOXP3^−^CD4^+^ and FOXP3^+^CD25^+^CD4^+^ cells per gram of tumor tissue were reduced following BO-112 treatment (Additional file 8: Figure S7C).

### Intratumoral BO-112 enlarges tumor-draining lymph nodes containing abundant CD8^+^ T cells

**Fig. 5:**
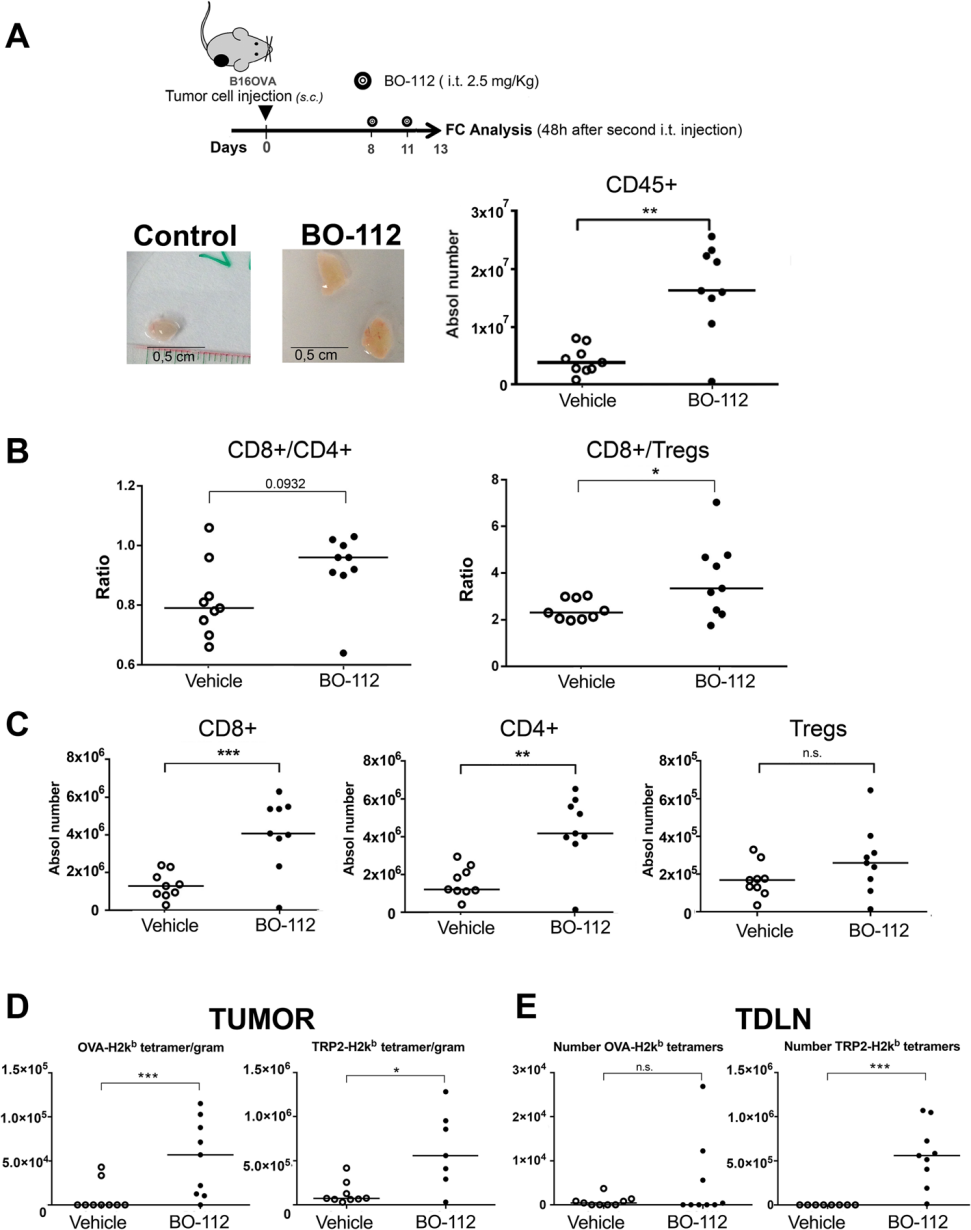
BO-112 intratumoral injection induces tumor-draining lymph node enlargement and increases CD8 T cells recognizing specific antigens. **a**. Scheme of experimental treatment showing representative size of TDLN and their total leukocyte content in the graph comparing mice treated intratumorally with BO-112 or control vehicle. **b** and **c**.: Analysis by flow cytometry of individual TDLN cell suspensions. **b**. CD8 to CD4 ratios and CD8/Treg ratios. **c**. represents the absolute number of the indicated T-cell subsets in TDLNs. **d**. Class I MHC tetramer stainings to identify T cells recognizing OVA-specific epitope and TRP-2 among CD8 T cells per gram of malignant tissue in mice bearing B16-OVA tumors. **e** Class I MHC tetramer stainings to identify the numbers OVA- and TRP2-specific CD8+ T cells in TDLN. Absolute numbers are provided for antigen-specific CD8 T cells. *P < 0.05, **P < 0.01***P < 0.001

Intratumoral release not only reaches the tumor microenvironment itself, but also drains to lymph nodes. In our hands, two intratumoral injections of BO-112 at therapeutic doses (Fig. 5a) in B16-OVA-tumor bearing mice resulted in prominent draining lymph node enlargements (Fig. 5b), as a result of an enhanced contents of CD45^+^ leukocytes. Again, CD8^+^/ CD4^+^ and CD8^+^/Treg ratios were markedly increased by treatment in most tumors, while non-draining lymph nodes remained normal (data not shown). Of interest, while numbers of effector CD8^+^ and CD4^+^ T cells rose, Tregs remained stable (Fig. 5c).

### Antitumor activity of intratumoral BO-112 requires IFNγ and correlates with increases in tumor-reactive CD8^+^ T cells

Results from tumor infiltrates suggested that an important component in the therapy was mediated by the immune system. To determine which cell subsets were involved in the BO-112 tumor growth delay, depletion experiments were performed in MC38 and B16-OVA tumors. Antitumor effects on MC38-derived tumors completely disappeared when CD8_β_^+^ T cells were depleted in vivo, while CD4+ T cells were dispensable (Additional file 9: Figure S8A and B). By contrast, single CD8 subset depletion induced only a partial loss of efficacy and single CD4+ and NK+ subset depletion had not impact in BO-112-mediated antitumor response in B16-OVA mouse models. (Additional file 10: Figure S9 A and B). Interestingly, triple depletion of NK1.1+, CD4+ and CD8+ had a major impact on BO-112 therapeutic effects (Additional file 10: Figure S9 B), although such loss of efficacy was not complete, indicating that other mechanisms are involved in BO-112-induced antitumor response. In addition, myeloid Gr-1+ cells were dispensable (Additional file 10: Figure S11 A and B). Depletions were verified in peripheral blood by flow cytometry (Additional file 9: Figure S8C and Additional file 10: Figure S9 C and D), and Gr-1 depletion was additionally verified in both blood and tumor (Additional file 11: Figure S10 C and D).

The immunotherapeutic effects mediated by CD8^+^ T cells are usually dependent on IFNγ and the critical contribution of this cytokine to intratumoral BO-112 efficacy was revealed in BO-112-treated B16-F10 tumors when IFNγ was systemically neutralized with a specific mAb (Additional file 11: Figure S10E).

Various tumor specificities of CD8^+^ T lymphocytes in TDLN and in the tumor microenvironment can be monitored with H-2k^b^ tetramers refolded with well-studied immunodominant CTL epitopes such as Ovalbumin (OVA) and TRP2 in B16-OVA, and gp70 in the MC38 model. In this regard, injections of BO-112 into B16-OVA tumors resulted in increased contents CD8^+^ T cells recognizing TRP-2 and OVA as a surrogate tumor antigen in tumors (Fig. 5d) and in TDLN (Fig. 5e).

In line with these findings in B16-OVA models, a remarkable increase of gp70-specific intratumor CD8+ T cells was found in MC38-tumor bearing mice treated intratumorally with BO-112 (Additional file 12 Figure 11).

### Intratumoral BO-112 induces an IFNα/β-related transcriptomic profile and type I interferon as well as cDC1 dendritic cells are required for antitumor effects

**Fig. 6:**
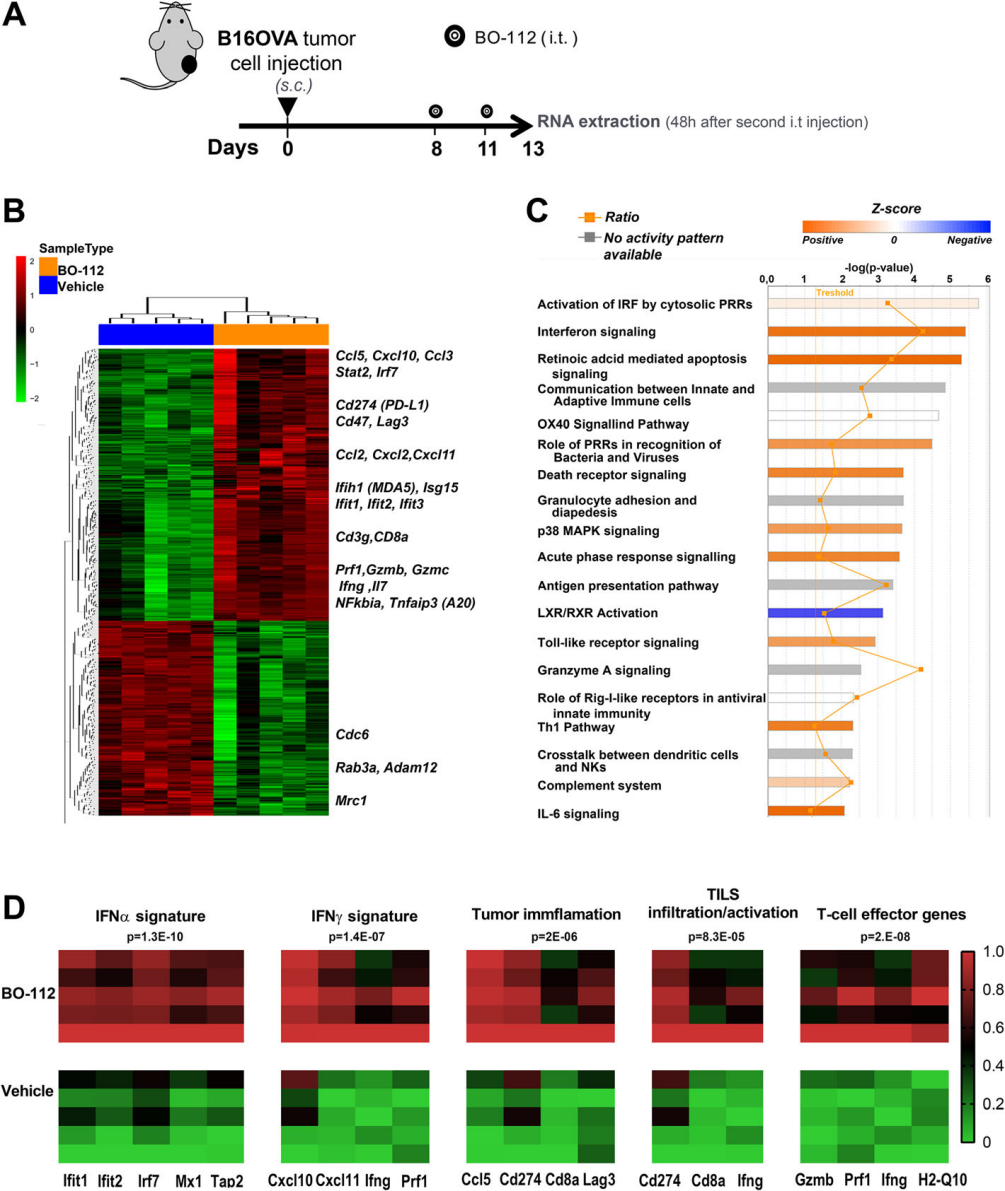
Intratumoral BO-112 induces potent type-I IFN-related transcriptomic changes. **a**. Mice bearing B16-OVA tumors were treated with intratumoral BO-112 or vehicle (*n* = 5 per group) and total RNA was extracted as indicated to be genome-wide analyzed by gene expression microarrays. Differentially expressed transcripts were obtained by Linear Models for Microarray Data (LIMMA) analysis (**b**). Hierarchical clustering of differentially expressed genes between both experimental conditions. Most relevant genes for immune functions are indicated as upregulated by BO-112. **c**. Top canonical pathways upregulated by BO-112 treatment as defined by Ingenuity Pathway Analysis of the differentially expressed transcripts. **d**. Heat map representing enrichment analyses of key previously described signatures for IFNα and IFNγ stimulation, for tumor cell infiltration and activation of TILs as well as T-cell effector-related transcripts

Previous reports have linked Poly I:C delivery to IFNα/β release [45, 46]. We genome-wide analyzed the mRNAs expressed in B16-OVA tumors 48 h following the second intratumoral BO-112 administration in comparison with control Vehicle (Fig. 6a and Additional file 13: Figure S12) and in B16-OVA cell cultures following 24 h incubation with BO-112 of Vehicle (Additional file 14: Figure S13).

**Fig. 7:**
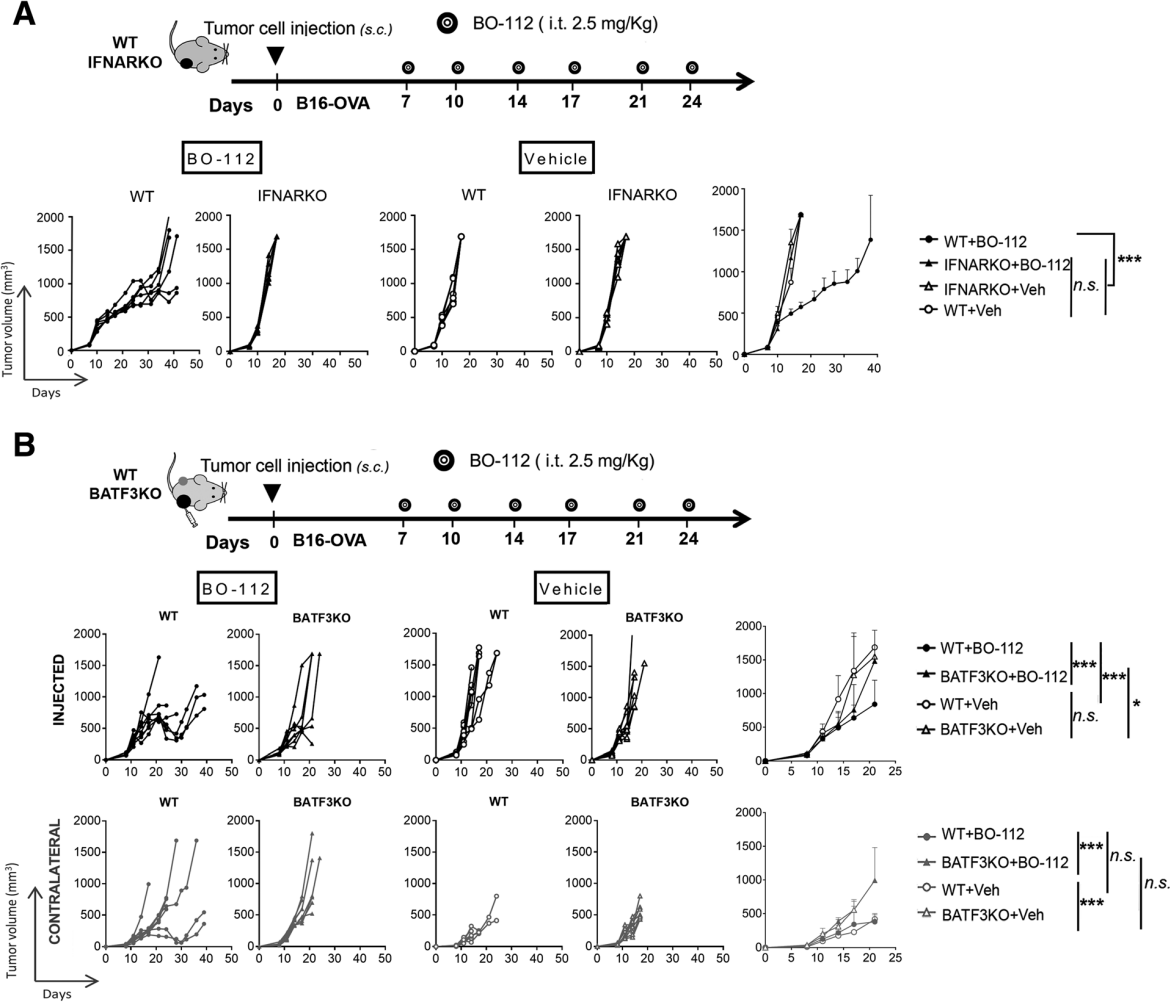
Antitumor response of intratumoral BO-112 is dependent on IFNα signaling and on Batf3-dependent Dendritic Cells. **a**. Tumor volume follow-up of WT and IFNARKO mice bearing B16-OVA tumors that were treated with intratumoral BO-112 or vehicle (*n* = 6 per group) as indicated in the diagram. Individual tumor volume and tumor volume means ± SD are shown. **b**. Tumor volume growth of WT or Batf3^−/−^ (BATF3KO) mice bearing two B16-OVA-derived tumors in which one was treated with BO-112 or vehicle (*n* = 6 per group) as indicated in the diagram. Tumor volume means ± SD are shown in graphs on the right. *P < 0.05, **P < 0.01***P < 0.001

As expected, a very clear differential gene expression profile was found upon intratumoral BO-112 administration, involving key immune-response genes whose expression rose in a clear-cut pattern, as shown in the hierarchical clustering of Fig. 6b and extended data in Additional file 15: Table S2. The differentially expressed gene set was significantly enriched in immune-response gene signatures involved in Interferon signaling and retinoic acid-mediated apoptosis (Fig. 7c and Additional file 16: Table S3). The BO-112-induced transcriptional profile was also enriched in previously reported gene signatures that suggest infiltration by activated immune cells and cytolytic activity [38, 39] (Fig. 6d). This gene expression pattern is comparable to that induced by poly I:C, since it best fits in *Ingenuity pathway analysis* (IPA) what has been described for cell exposure to Poly I:C, as expected from stimulation of TLR3 and cytosolic pattern recognition receptors (PRRs) (Additional file 17: Table S4).

Key genes involved in IRF activation by cytosolic PRRs, IFN signaling, retinoic acid-mediated apoptosis were among the 254 differentially expressed genes shared between in vitro and in vivo experiments (Additional file 14: Figure S13), while other key immunoregulatory genes were upregulated by BO-112 in B16-OVA cell cultures (Additional file 14: Figure S13B and Additional file 18: Table S5).

All these effects on the transcriptomic profiles speak of an excellent mimicry of viral infection in the BO-112-injected tumor microenvironment conducive to the enhancement of anti-tumor cytotoxic T-cell responses.

As suggested by the observed induced expression of IFN-I, IFNARKO mice bearing B16-OVA tumors were found not to respond to intratumor BO-112, thereby providing evidence for the key role of IFNα/β signaling for the antitumor response induced by BO-112 (Fig. 7a). Tumor antigen crosspriming is known to be up-regulated by type I IFN and TLR3 function. CD8 T-cell crosspriming is critically mediated by so called conventional type-I dendritic cells (cDC1) that are absent in BATF3^−/−^ mice [33]. Experiments of intratumoral treatment with BO-112 performed in BATF-3^−/−^ mice bearing two B16-OVA tumors (in which one was left uninjected) showed that this cDC-1 subset is crucial for the therapeutic response to local BO-112, since the progression delay mediated by in situ delivery of BO-112 was completely lost when compared to wild type mice (Fig. 7b).

## Discussion

In this study we have explored BO-112, a nanoparticled form of Poly I:C administered via the intratumoral route. Intratumoral delivery of immunotherapy came to age with the FDA approval of the HSV-1 vector T-vec for locally advanced or metastatic melanoma [47]. However viral vectors are immunogenic and often encode pathogenic factors that downregulate cellular immunity. Viral-like nanoparticles such as BO-112 have the theoretical advantage of lacking immunogenicity, thus theoretically permitting limitless dose repetition. Moreover, they do not need the logistical and safety cautions that must be taken into account with viruses or modified recombinant viruses. In addition, a very strong dsRNA immunostimulatory profile proceeds unchecked by any interference from counteracting viral immunosuppressive proteins.

In our hands, BO-112 is therapeutically active when used intratumorally, showing anti-tumoral efficacy in experimental settings in which intratumoral Poly I:C did not. This offers advantages since doses can be kept far from toxic thresholds, while achieving at least local antitumor effects. Interestingly, similar subcutaneous dosing of BO-112 shows no efficacy as compared to intratumoral release. Consistent with other forms of Poly I:C, intratumoral BO-112 is safe in mice and achieves marked tumor control of injected transplanted tumors including poorly immunogenic variants.

Mechanistic studies dissecting the mode of action result in the following model: first, BO-112 induces cell death in a small fraction of tumor cells in the context of alarmins denoting stressful non-programmed cell death [42]. This may result in tumor antigen release and crosspresentation by professional antigen-presenting cells [48] including BATF-3 dependent c-DC1. At the same time, strong IFNα/β release and other proinflammatory mediators act as a local adjuvant in this context of in-situ vaccination [11, 49]. As a consequence, a tumor-specific CD8 immune response is mounted or augmented to the point of controlling tumor progression, both in the locally injected lesion and to some extent in distantly implanted tumor nodules. This is consistent with increases of tumor-specific CD8 T cells in the tumor microenvironment and TDLNs.

Mechanisms aside adaptive immunity are operational in the treatment as seen upon simultaneous depletion of T and NK lymphocytes. On the one hand there are direct cytotoxic effects to tumor cells and on the other there might be effects on the functionality of innate immune cells other than NK lymphocytes.

Other TLR [1] and STING [50] agonists are being developed for intratumoral injection in the clinic (for instance, the following ongoing clinical trials registered in clinicaltrial.gov: NCT02927964; NCT02423863; NCT02501473; NCT03172936). However, the induction of immunogenic cell death to a certain level could be an important advantage in the case of BO-112. It remains to be seen which, or which combination, of agonists to PRRs behaves as the most beneficial when injected intratumorally.

In the era of checkpoint inhibitors, combinations of local agents and systemic immunomodulatory mAbs make much sense [51]. In the case of intratumoral BO-112, we observe some additive effects with anti-PD-L1 and anti-CD137 mAbs, that were not truly synergistic. In this regard, clinical reports on results of the anti-PD-1 mAb combinations with other TLR agonists such as G100, CpG oligonucleotides and STING agonists given locally are eagerly expected. This is also the case of intratumoral oncolytic virus T-vec that in combination with pembrolizumab has shown remarkable responses in metastatic melanoma patients [52], being pending of the results of the randomized clinical trial MASTERKEY 265 in combination with pembrolizumab versus pembrolizumab alone (NCT02263508). In the case of BO-112, the expression of CD137 and PD-1/PD-L1 was increased on tumor infiltrating T and NK cells, a fact that hinted at the potential combinability of the dual local and systemic approach.

All things considered, we have observed that intratumoral BO-112 is active in local cancer immunotherapy. It remains to be studied what would be the best combination regimen, but for the time being BO-112 is combined in the clinic with anti-PD-1 mAbs, since our results have been conductive to an ongoing clinical trial (NCT02828098) which is testing the safety and clinical activity of intratumoral BO-112 either as a single agent or in combination with nivolumab or pembrolizumab checkpoint inhibitors. The transcriptomic gene-expression profile induced by BO-112 in engrafted mouse tumors offers potential for pharmacodynamics biomarkers and, as a consequence, RNA expression assessments are being carried out in pre- and post-treatment biopsies of injected tumors taken from patients on trial.

## Conclusions

Nanoplexed Poly I:C (BO-112) when locally injected induces immunogenic cell death in a fraction of tumor cells and exerts potent antitumor activity via strong induction of type I interferon and CD8 T-cell infiltrates in the tumor microenvironment. As a result of these findings intratumoral BO-112 is undergoing phase I/II clinical trials.

## Additional files

Additional file 1: **Table S1.** Flow cytometry mAbs and other staining dyes employed for multiparametric flow cytometry analyses. (XLSX 12 kb)

Additional file 2: **Figure S1.** A representative BO-112 intensity size distribution is presented, that was determined by Dynamic Light Scattering (DLS), a non-invasive technique for measuring the size of particles in suspension. Above of the DLS graph there is a cartoon representing the postulated structure of a BO-112 nanocomplex. (TIF 513 kb)

Additional file 3: **Figure S2.** BO-112 cytotoxic effects on human tumor cell lines. A. Cell viability experiments as in Fig. 1a showing the effects of various doses of BO-112 or Poly I:C on human tumor cell lines representing melanoma, colon cancer and breast cancer. B. Cell viability of B16-OVA, MC38, HCT 116 and HT-29 tumor cell lines upon incubation with increasing amounts of BO-112 for 24 and 48 h. Cell viability was assessed by MTS assay. % Viability is referred to untreated cells. C**.** Early and late apoptosis assessment induced by BO-112 in two representative human tumor cell lines measured by flow cytometry as in Fig. 2a. (TIF 1168 kb)

Additional file 4: **Figure S3.** Intratumor delivery of polyethylenimine is unable to induce therapeutic effects. A. xCELLigence experiments as in Fig. 1a showing B16-OVA cell viability upon in vitro incubation with BO-112 or PEI in equivalent amounts as present in each BO-112 dose. B. Timeline showing the treatment schedule of intratumoral administrations of BO-112 or PEI in B16-OVA models. Mice were injected subcutaneously with B16-OVA at day 0 (5 × 10^5^ cells) in the right flank. When tumor size reached 80–100 mm^3^, animals were treated with PEI or BO-112 by injection into the tumor nodules (i.t). Plots show individual volume (length x width^2^/2) for control (vehicle) and PEI and BO-112 treated mice. ***P < 0.001. (TIF 4613 kb)

Additional file 5: **Figure S4.** BO-112 therapeutic effects require intratumoral administration. A. Timeline showing the schedule of experimental treatment. All mice were injected subcutaneously with B16-OVA or MC38 murine colon carcinoma cells at day 0 (5 × 10^5^ cells) in the right flank. After mice randomization (when tumor size reached 80–100 mm^3^), animals were treated with BO-112 by injection into the tumor nodules (i.t) or by subcutaneous injection in the left flank (s.c). Plots show individual volume (length x width^2^/2) for control (vehicle) and BO-112 treated mice, following i.t and s.c routes of drug administration as indicated. ***P < 0.001 (TIF 5096 kb)

Additional file 6: **Figure S5.** Gating strategy for flow cytometry analyses to study tumor infiltration in in vivo experiments. Flow cytometry plots of a representative sample showing the gating strategy for TIL analysis. (TIF 1065 kb)

Additional file 7: **Figure S6.** Expression of CD137, PD-1 and PD-L1 on infiltrating lymphocytes from BO-112-treated B16-OVA tumors. A. Flow cytometry analysis of the intensity of expression of surface PD-1, CD137 and PD-L1 on gated CD4^+^, CD8^+^ and NK lymphocytes comparing tumors treated with BO-112 or vehicle. B**.** Percentage of CD8^+^ cells coexpressing PD-1 and CD137 and their density per gram of tumor as analyzed following intratumoral treatment with BO-112 or control vehicle. **P < 0.01***P < 0.001 (TIF 1114 kb)

Additional file 8: **Figure S7.** BO-112 intratumoral injection enhances T lymphocyte infiltrates of MC38 tumors. A. Schematic representation of the experiments to generate cell suspensions of MC38 tumors that were analyzed by flow cytometry. B. Flow cytometry frequencies of PD-1^+^, CD137^+^ and PD-1^+^CD137^+^ double positive CD8+ in the CD8+ T cell population infiltrating MC38 tumors after two intratumoral injections of BO-112, and absolute numbers of CD8^+^ T cells per gram of tumor. C. Absolute number per gram of CD4 and CD25 + CD4+ Tregs per gram of tumor. *P < 0.05**P < 0.01 (TIF 3622 kb)

Additional file 9: **Figure S8.** CD8 depletion abrogate therapeutic effects of BO-112 intratumoral delivery in MC38-tumor bearing mice. A. Schematic representation of experiments on mice bearing MC38-derived tumors for lymphocyte subset depletion. B. Individual follow-up upon treatment with BO-112 or vehicle in mice depleted of CD8 or CD4 T cells by specific monoclonal antibodies. C. CD4+ and CD8+ depletion validation in peripheral blood of a representative group of animals was analyzed by flow cytometry during the experimental procedure. **P < 0.01***P < 0.001. (TIF 1913 kb)

Additional file 10: **Figure S9.** CD8, CD4 and NK depletion in B16-OVA tumor-bearing mice treated with intratumoral BO-112. A. Schematic representation of experiments on B16-OVA-tumor bearing mice that were depleted from the indicated lymphocyte subsets. B. Individual tumor volume follow-up in groups of mice intratumorally treated with with BO-112 or vehicle and depleted from CD8+, CD4+ or NK1.1+ cells separately or concomitantly. Lymphocyte cell subsets were selectively depleted by specific monoclonal antibodies. The corresponding statistical comparisons are summarized below the graphs. C. Representative dot plots of NK1.1+, CD4+ and CD8+ lymphocyte depletions as assessed in peripheral blood analyzed by flow cytometry during the experimental procedure. In D levels of depletion achieved in individual mice are shown. *P < 0.05 **P < 0.01 ***P < 0.001. (TIF 2355 kb)

Additional file 11: **Figure S10.** Gr-1 depletion and IFNγ neutralization in tumor-bearing mice treated with intratumoral BO-112. A. Schematic representation of experiments on B16-OVA-tumor bearing mice that were treated for Gr-1 depletion. B. Tumor volume follow-up in mice depleted of Gr-1 cells by a specific monoclonal antibody and intratumorally treated with with BO-112 or vehicle. C. Gr-1 depletion validation in B16-OVA tumors analyzed by flow cytometry on day 7 coinciding with the first intratumoral injection of BO-112. D. Gr-1+ depletion validation in peripheral blood performed as in C. E. Experiments in B16-F10 melanoma-bearing mice treated with intratumoral BO-112 or vehicle recording individual tumor sizes. When indicated, mice were given neutralizing anti-IFNγ mAb or isotype control. **P < 0.01***P < 0.001. (TIF 2920 kb)

Additional file 12: **Figure S11.** Increases of tumor-specific CD8+ T cells recognizing the gp70 tumor antigen following BO-112 injections into MC38 tumors. A. Diagram showing treatment schedule for MHC-I pentamer staining to identify gp70-specific CD8 T cells in mice bearing MC38 tumors. Frequencies of antigen-specific CD8 T cells infiltrating tumors (B.) and TDLNs (C.) are shown. *P < 0.05. (TIF 2584 kb)

Additional file 13: **Figure S12.** Volcano Plot highlighting top differentially expressed genes (as per FC) in BO-112-treated B16-OVA tumors. RNA derived from B16-OVA tumors treated with intratumoral BO-112 or vehicle as indicated in Fig. 6 was analyzed by expression microarrays. Differentially expressed genes with a │logFC│ > 1 and *p* > 0.01 are considered differentially expressed in BO-112-treated B16-OVA tumors. (TIF 581 kb)

Additional file 14: **Figure S13.** Key immunoregulatory genes differentially expressed upon BO-112 intratumoral administration are also induced in B16-OVA cultures incubated with BO112. The B16-OVA cell line was incubated either with BO-112 or vehicle for 24 h and its RNA was genome wide analyzed with gene-expression microarrays. A. Venn diagram showing the differentially expressed genes that were shared with both in vitro and in vivo procedures (top) and top 19 canonical pathways in predicted by Ingenuity Pathway analysis (bottom). B. Hierarchical clustering of differentially expressed genes in B16-OVA after BO-112 incubation. (TIF 964 kb)

Additional file 15: **Table S2.** Differentially expressed genes obtained upon BO-112 intratumoral administration. Mice bearing B16-OVA tumors were treated with intratumoral BO-112 or vehicle as indicated in Fig. 6, and total RNA was extracted and analyzed by expression microarrays. Genes were selected as significant using a B-statistic cut-off (B > 0). (XLSX 195 kb)

Additional file 16: **Table S3.** Top canonical differentially regulated pathways induced by BO-112 intratumoral administration. Pathways from differentially expressed genes upon BO-112 intratumoral administration (selected as significant using a B-statistic cut-off B > 0) were identified by Ingenuity Pathway Analysis. (XLS 35 kb)

Additional file 17: **Table S4.** Top 30 Upstream Regulators predicted to promote the differentially expression profile induced by BO-112 intratumoral administration. Upstream Regulators from differentially expressed genes upon BO-112 intratumoral administration (selected as significant using a B > 0 cut-off) were identified by Ingenuity Pathway Analysis. (XLSX 17 kb)

Additional file 18: **Table S5.** Differentially expressed genes induced by BO-112 in B16-OVA in vitro. B16-OVA cell line was incubated either with BO-112 or vehicle for 24 h and its RNA was genome wide analyzed with gene-expression microarrays. Genes were selected as significant using a B > 0 cut-off. (XLSX 241 kb)

## Abbreviations

cDC1: conventional type-I dendritic cells
GEO: Gene Expression Omnibus
IFN-I: Type-I interferon
IFNγ: Interferon-γ
IPA: Ingenuity Pathway Analysis
LIMMA: Linear Models for Microarray Data
PRRs: cytosolic pattern recognition receptors
RMA: Robust Multichip Average algorithm
TDLN: Tumor-draining lymph nodes
