# Supplementary figures and images for: Immunotherapeutic effects of intratumoral nanoplexed poly I:C
Source: J Immunother Cancer. 2019 May 2;7:116. doi: 10.1186/s40425-019-0568-2 (PMC6498680; doi:10.1186/s40425-019-0568-2)

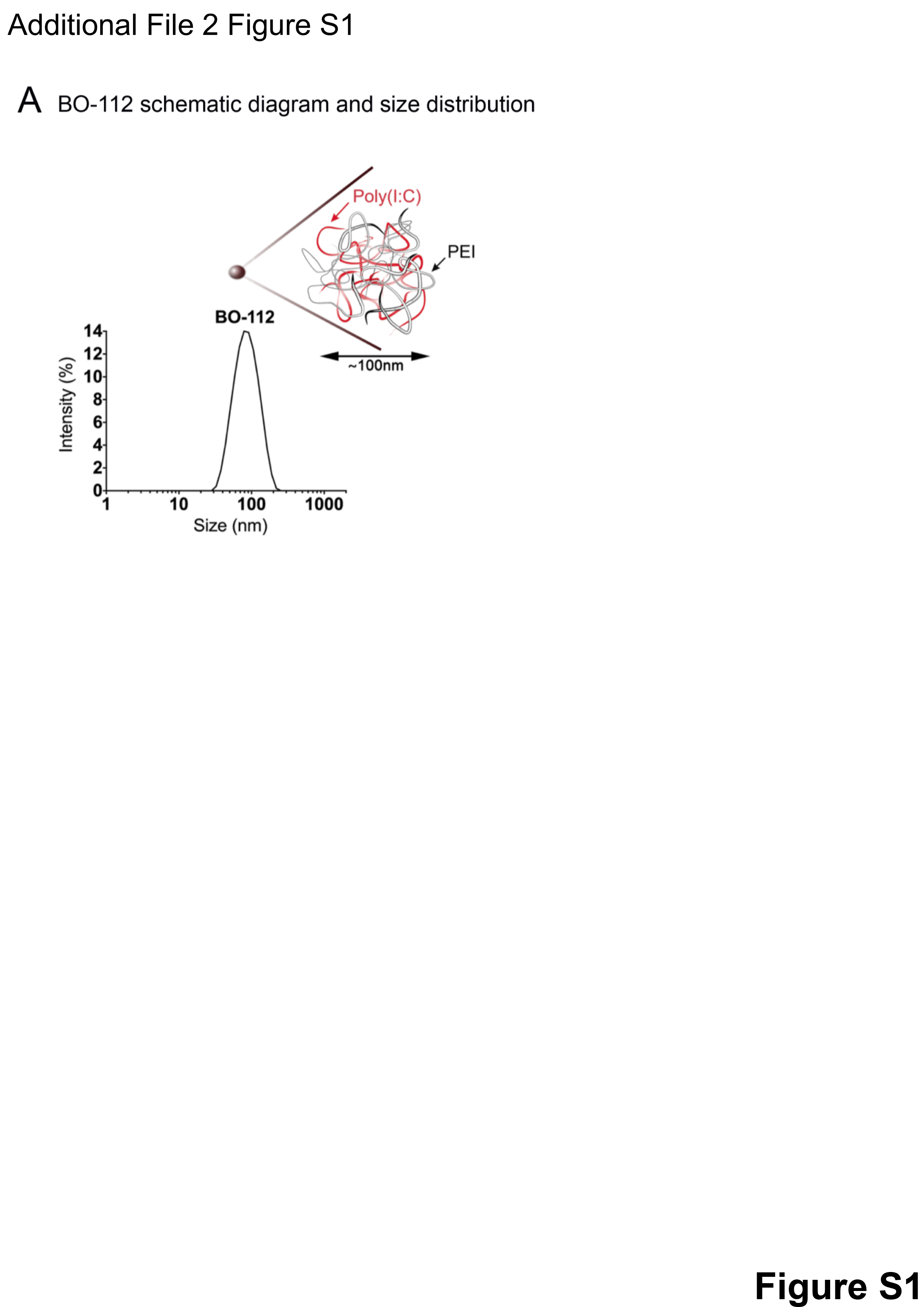

Supplement: Supplementary file 2 — Figure S1. A representative BO-112 intensity size distribution is presented, that was determined by Dynamic Light Scattering (DLS), a non-invasive technique for measuring the size of particles in suspension. Above of the DLS graph there is a cartoon representing the postulated structure of a BO-112 nanocomplex. (TIF 513 kb) [file 40425_2019_568_MOESM2_ESM.tif]

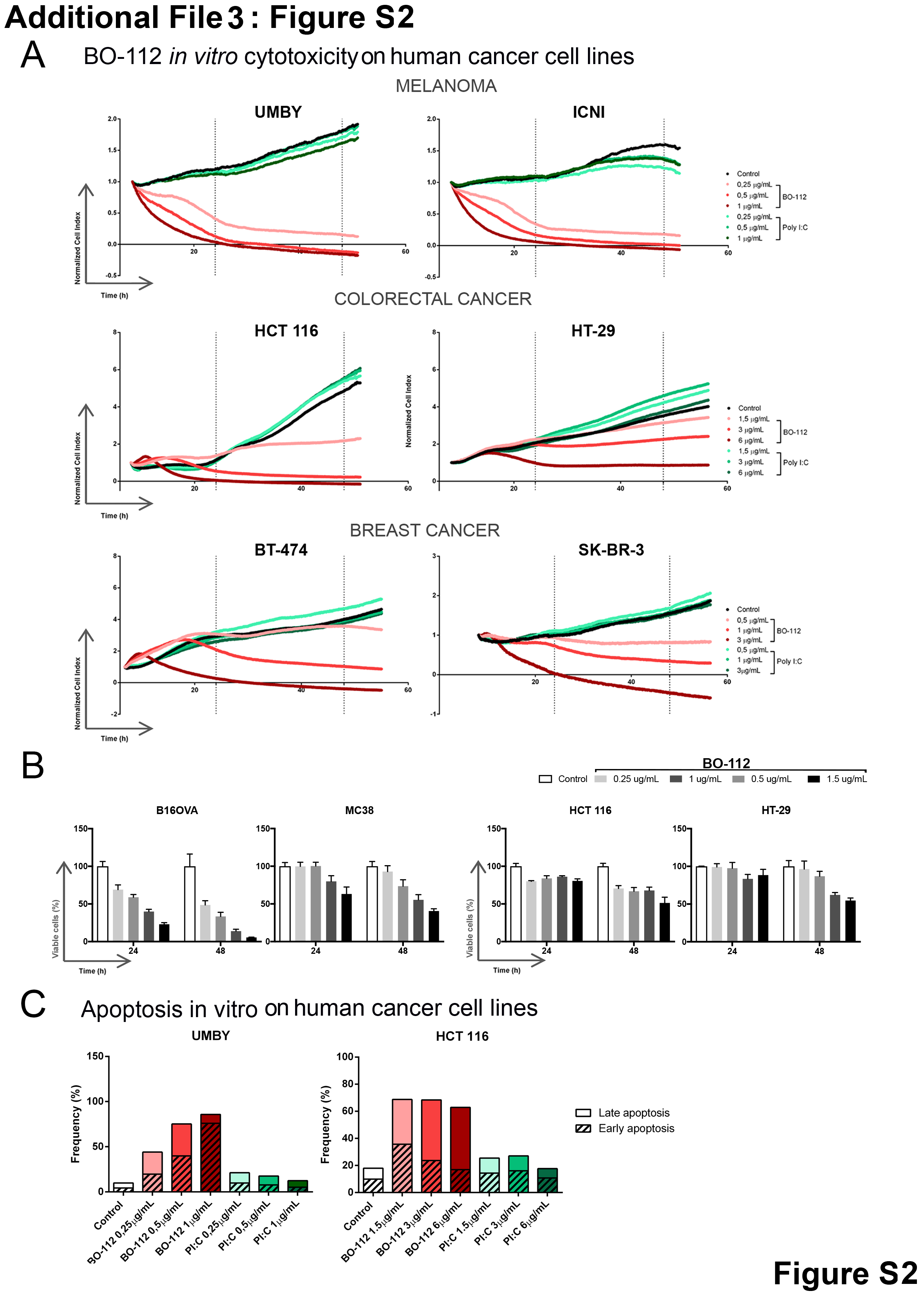

Supplement: Supplementary file 3 — Figure S2. BO-112 cytotoxic effects on human tumor cell lines. A. Cell viability experiments as in Fig. 1a showing the effects of various doses of BO-112 or Poly I:C on human tumor cell lines representing melanoma, colon cancer and breast cancer. B. Cell viability of B16-OVA, MC38, HCT 116 and HT-29 tumor cell lines upon incubation with increasing amounts of BO-112 for 24 and 48 h. Cell viability was assessed by MTS assay. % Viability is referred to untreated cells. C. Early and late apoptosis assessment induced by BO-112 in two representative human tumor cell lines measured by flow cytometry as in Fig. 2a. (TIF 1168 kb) [file 40425_2019_568_MOESM3_ESM.tif]

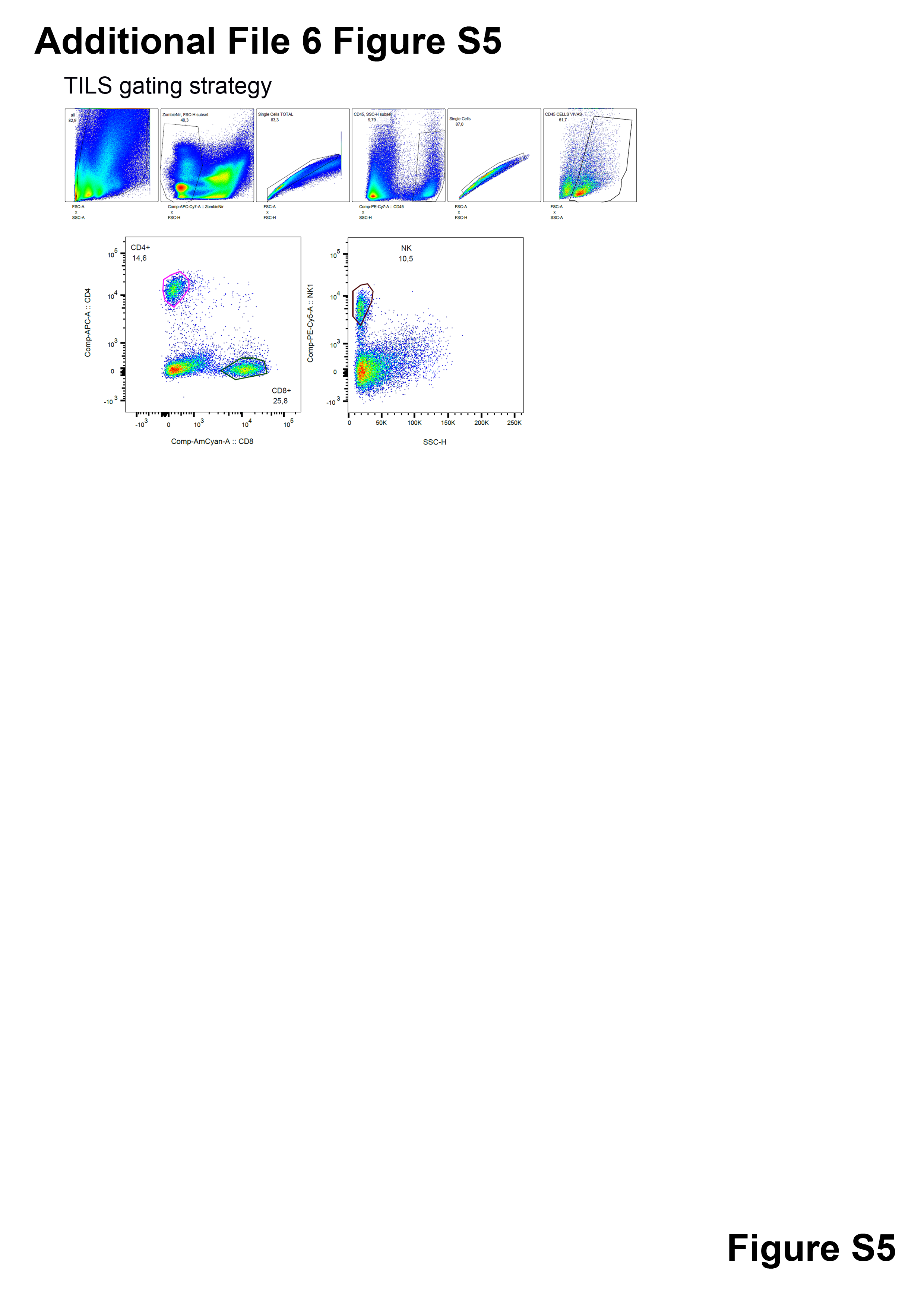

Supplement: Supplementary file 6 — Figure S5. Gating strategy for flow cytometry analyses to study tumor infiltration in in vivo experiments. Flow cytometry plots of a representative sample showing the gating strategy for TIL analysis. (TIF 1065 kb) [file 40425_2019_568_MOESM6_ESM.tif]

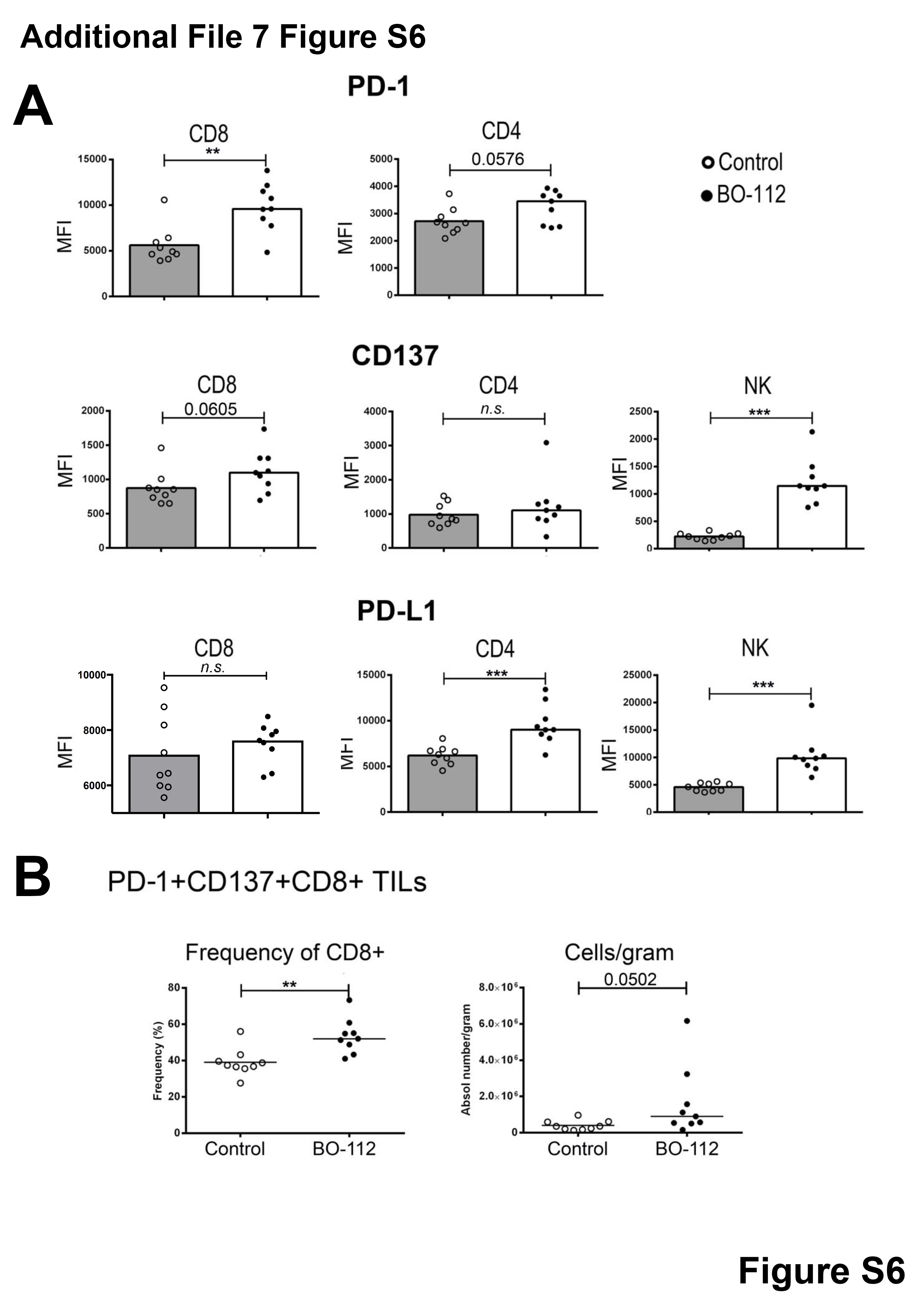

Supplement: Supplementary file 7 — Figure S6. Expression of CD137, PD-1 and PD-L1 on infiltrating lymphocytes from BO-112-treated B16-OVA tumors. A. Flow cytometry analysis of the intensity of expression of surface PD-1, CD137 and PD-L1 on gated CD4+, CD8+ and NK lymphocytes comparing tumors treated with BO-112 or vehicle. B. Percentage of CD8+ cells coexpressing PD-1 and CD137 and their density per gram of tumor as analyzed following intratumoral treatment with BO-112 or control vehicle. **P < 0.01***P < 0.001 (TIF 1114 kb) [file 40425_2019_568_MOESM7_ESM.tif]

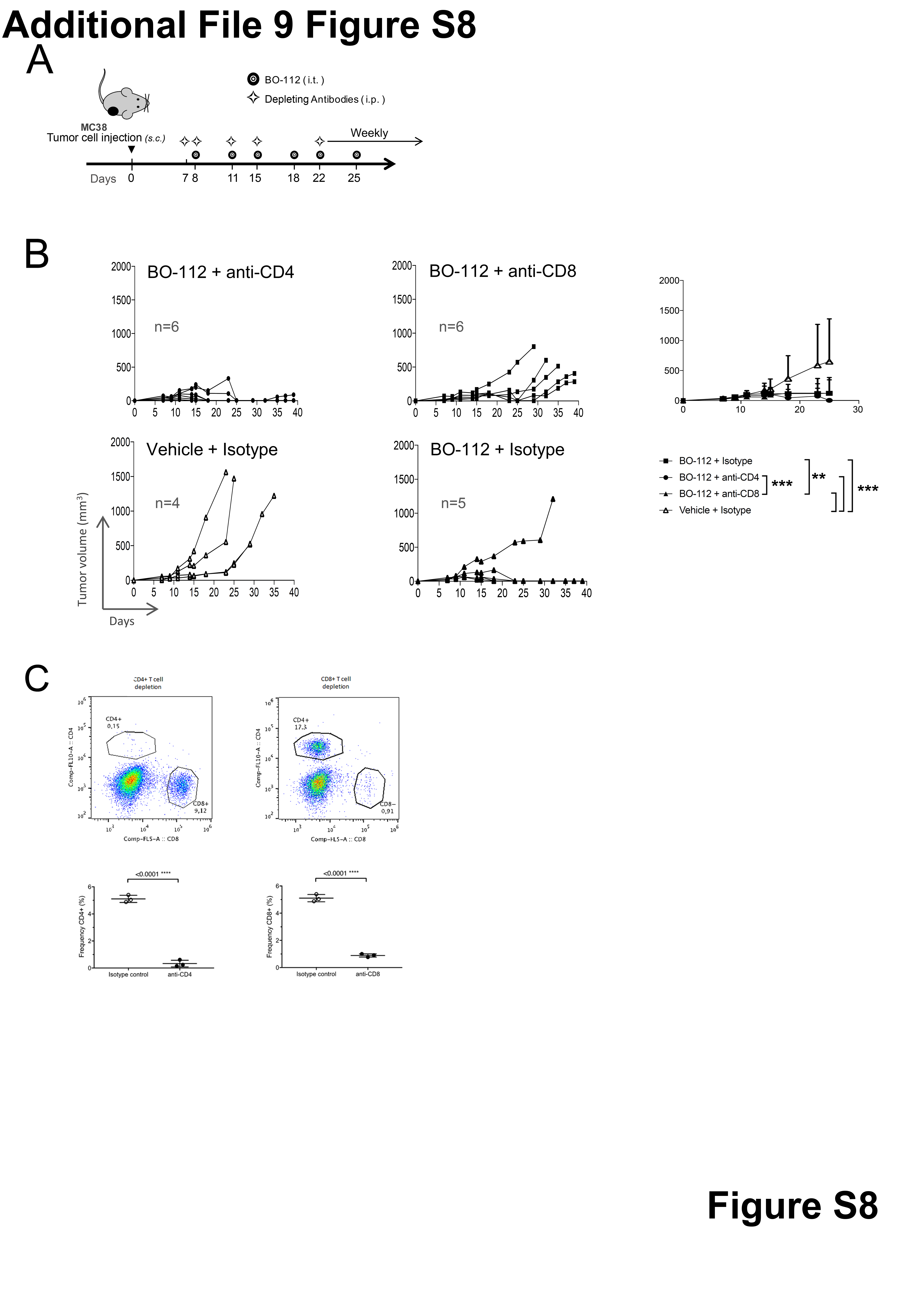

Supplement: Supplementary file 9 — Figure S8. CD8 depletion abrogate therapeutic effects of BO-112 intratumoral delivery in MC38-tumor bearing mice. A. Schematic representation of experiments on mice bearing MC38-derived tumors for lymphocyte subset depletion. B. Individual follow-up upon treatment with BO-112 or vehicle in mice depleted of CD8 or CD4 T cells by specific monoclonal antibodies. C. CD4+ and CD8+ depletion validation in peripheral blood of a representative group of animals was analyzed by flow cytometry during the experimental procedure. **P < 0.01***P < 0.001. (TIF 1913 kb) [file 40425_2019_568_MOESM9_ESM.tif]

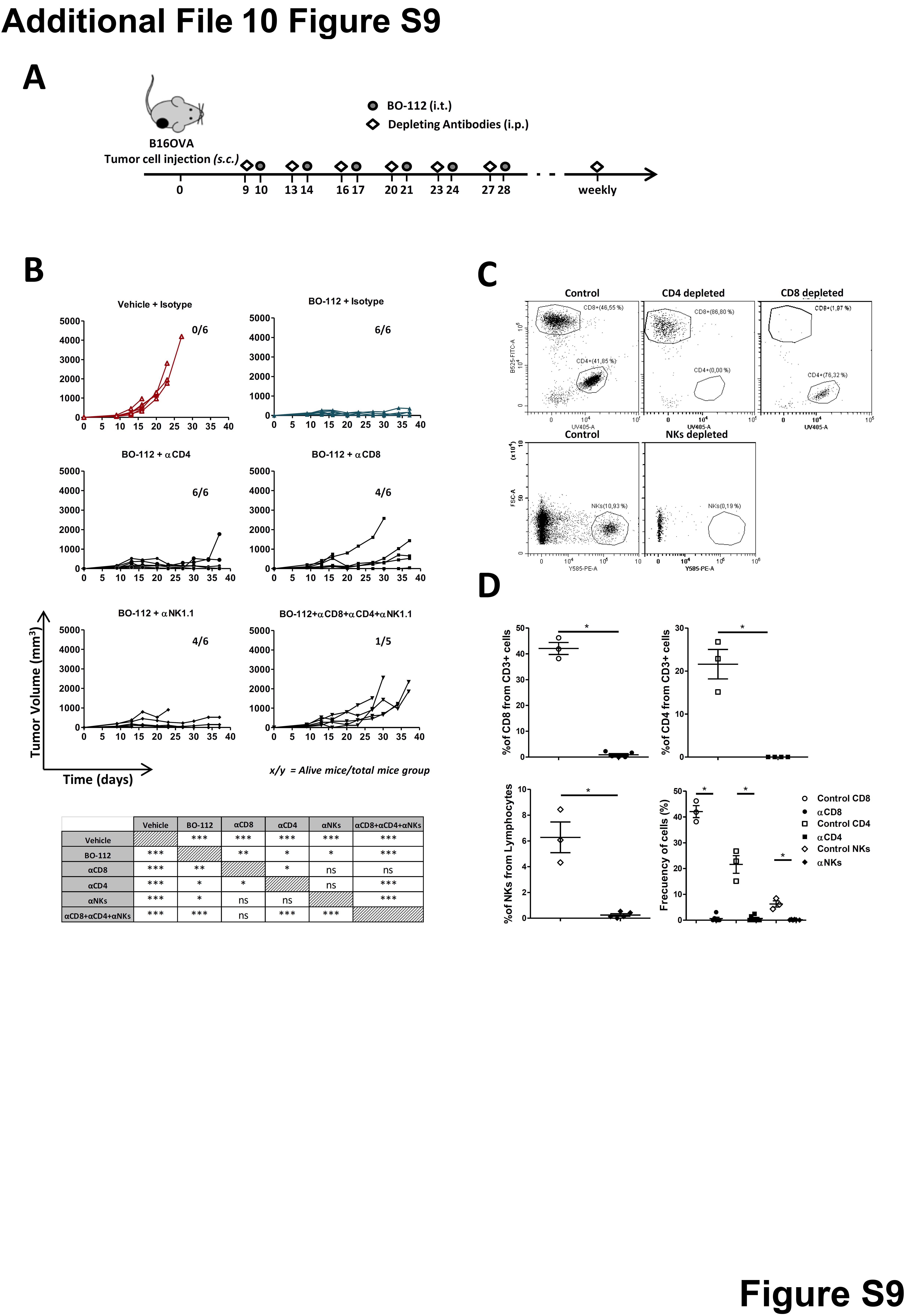

Supplement: Supplementary file 10 — Figure S9. CD8, CD4 and NK depletion in B16-OVA tumor-bearing mice treated with intratumoral BO-112. A. Schematic representation of experiments on B16-OVA-tumor bearing mice that were depleted from the indicated lymphocyte subsets. B. Individual tumor volume follow-up in groups of mice intratumorally treated with with BO-112 or vehicle and depleted from CD8+, CD4+ or NK1.1+ cells separately or concomitantly. Lymphocyte cell subsets were selectively depleted by specific monoclonal antibodies. The corresponding statistical comparisons are summarized below the graphs. C. Representative dot plots of NK1.1+, CD4+ and CD8+ lymphocyte depletions as assessed in peripheral blood analyzed by flow cytometry during the experimental procedure. In D levels of depletion achieved in individual mice are shown. *P < 0.05 **P < 0.01 ***P < 0.001. (TIF 2355 kb) [file 40425_2019_568_MOESM10_ESM.tif]

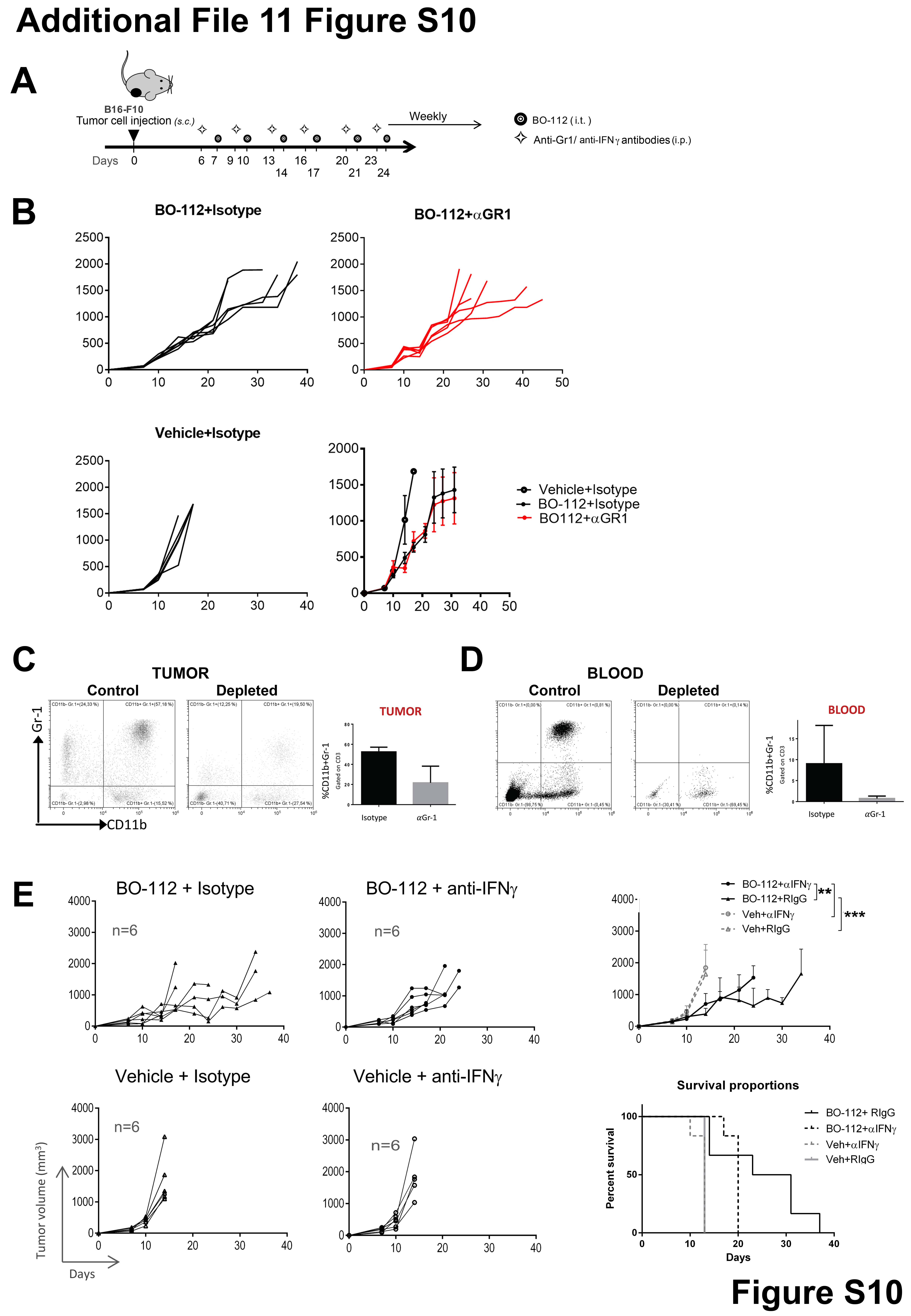

Supplement: Supplementary file 11 — Figure S10. Gr-1 depletion and IFNγ neutralization in tumor-bearing mice treated with intratumoral BO-112. A. Schematic representation of experiments on B16-OVA-tumor bearing mice that were treated for Gr-1 depletion. B. Tumor volume follow-up in mice depleted of Gr-1 cells by a specific monoclonal antibody and intratumorally treated with with BO-112 or vehicle. C. Gr-1 depletion validation in B16-OVA tumors analyzed by flow cytometry on day 7 coinciding with the first intratumoral injection of BO-112. D. Gr-1+ depletion validation in peripheral blood performed as in C. E. Experiments in B16-F10 melanoma-bearing mice treated with intratumoral BO-112 or vehicle recording individual tumor sizes. When indicated, mice were given neutralizing anti-IFNγ mAb or isotype control. **P < 0.01***P < 0.001. (TIF 2920 kb) [file 40425_2019_568_MOESM11_ESM.tif]

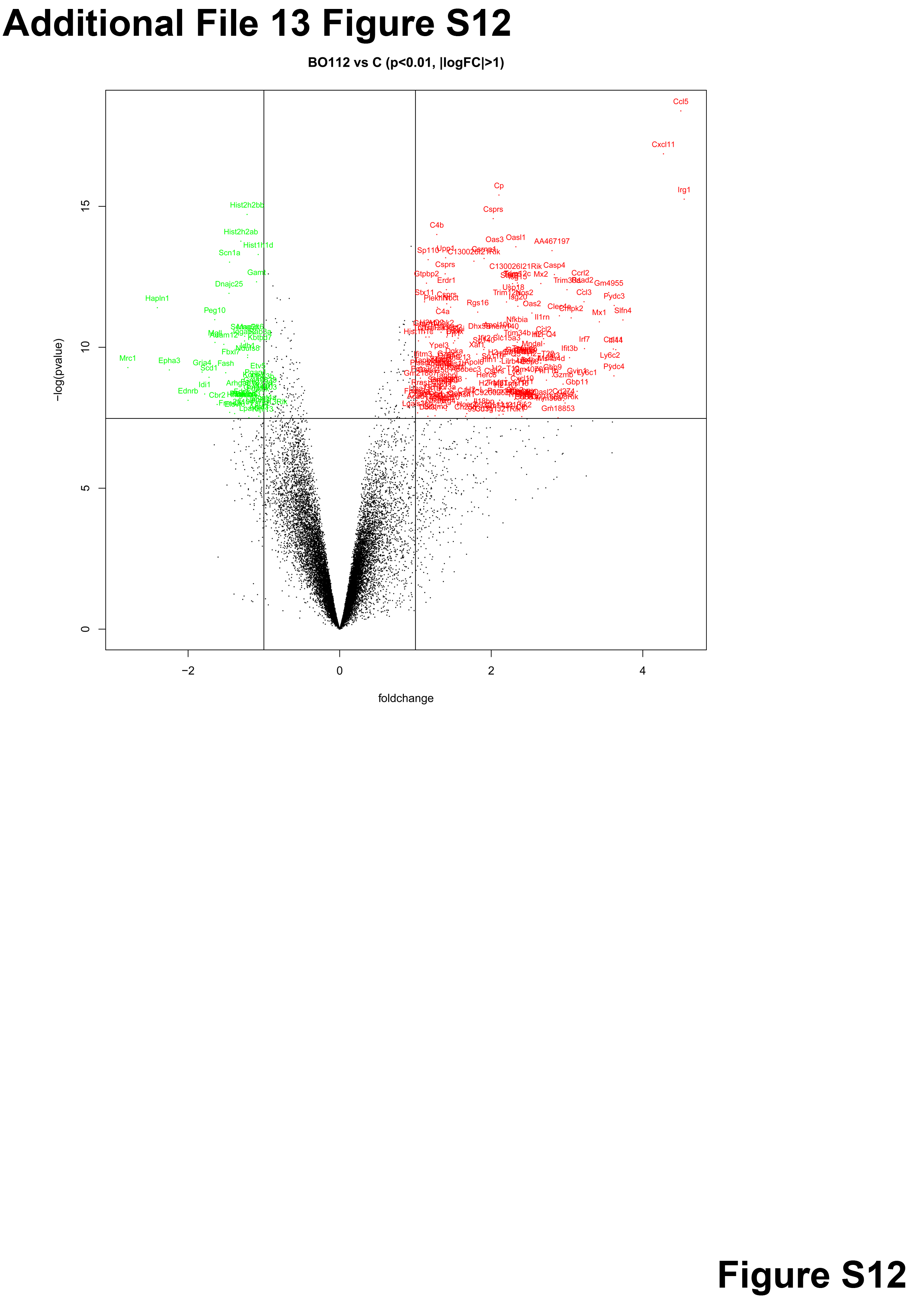

Supplement: Supplementary file 13 — Figure S12. Volcano Plot highlighting top differentially expressed genes (as per FC) in BO-112-treated B16-OVA tumors. RNA derived from B16-OVA tumors treated with intratumoral BO-112 or vehicle as indicated in Fig. 6 was analyzed by expression microarrays. Differentially expressed genes with a │logFC│ > 1 and p > 0.01 are considered differentially expressed in BO-112-treated B16-OVA tumors. (TIF 581 kb) [file 40425_2019_568_MOESM13_ESM.tif]

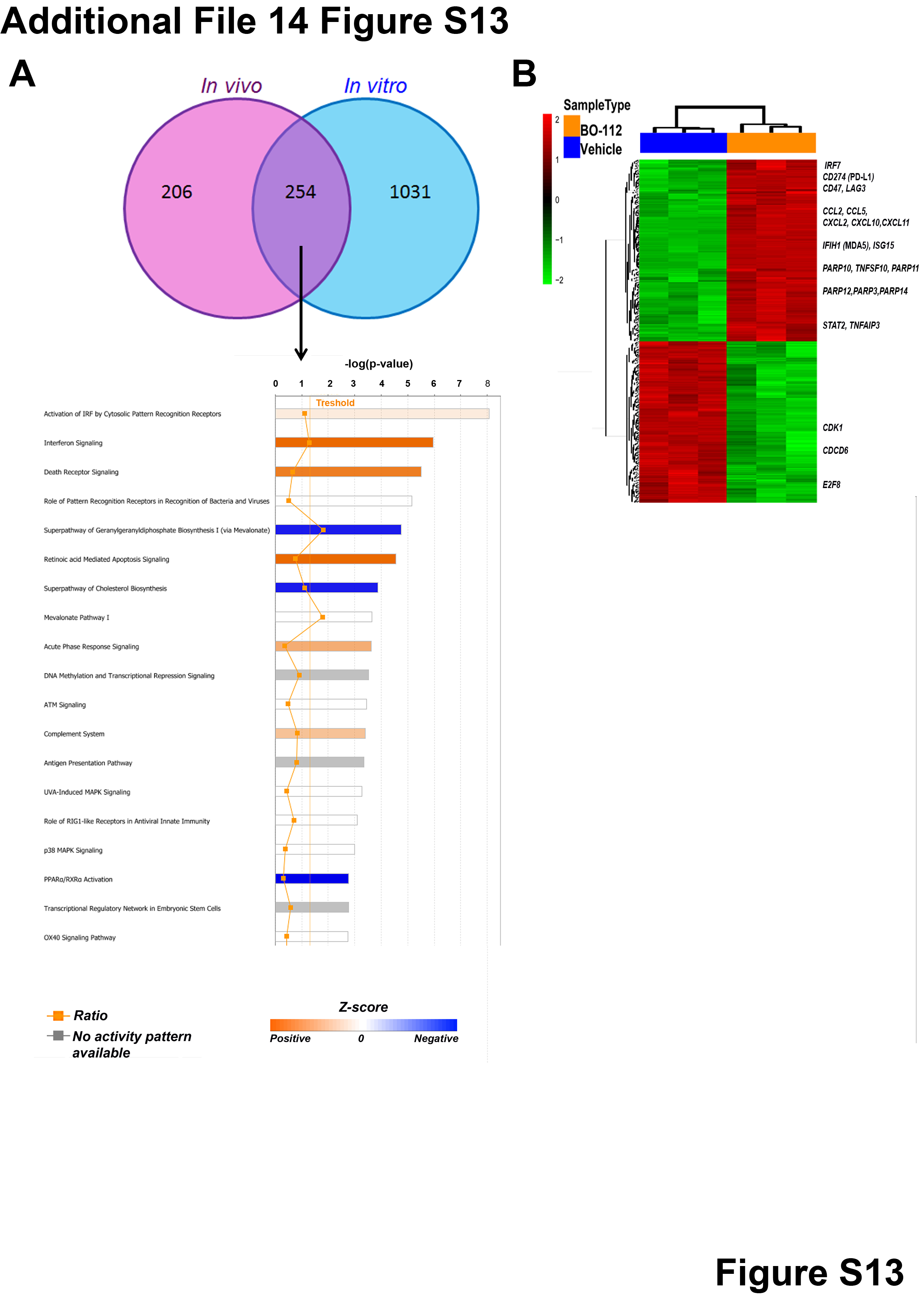

Supplement: Supplementary file 14 — Figure S13. Key immunoregulatory genes differentially expressed upon BO-112 intratumoral administration are also induced in B16-OVA cultures incubated with BO112. The B16-OVA cell line was incubated either with BO-112 or vehicle for 24 h and its RNA was genome wide analyzed with gene-expression microarrays. A. Venn diagram showing the differentially expressed genes that were shared with both in vitro and in vivo procedures (top) and top 19 canonical pathways in predicted by Ingenuity Pathway analysis (bottom). B. Hierarchical clustering of differentially expressed genes in B16-OVA after BO-112 incubation. (TIF 964 kb) [file 40425_2019_568_MOESM14_ESM.tif]
